# Urea‐Formaldehyde Resin Confined Silicon Nanodots Composites: High‐Performance and Ultralong Persistent Luminescence for Dynamic AI Information Encryption

**DOI:** 10.1002/advs.202522820

**Published:** 2026-01-20

**Authors:** Yulu Liu, Lei Cao, Lele Gao, Panyong Wang, Qiannan You, Xinpei Pang, Li Li, Mingzheng Jia, Wen‐Fei Dong, Minghui Zan

**Affiliations:** ^1^ Department of Biomaterials and Stem Cells Suzhou Institute of Biomedical Engineering and Technology Chinese Academy of Science (CAS) Suzhou P. R. China; ^2^ School of Biomedical Engineering (Suzhou) Division of Life Sciences and Medicine University of Science and Technology of China Hefei P. R. China; ^3^ Anhui Key Laboratory of Biomedical Materials and Chemical Measurement Key Laboratory of Functional Molecular Solids Ministry of Education College of Chemistry and Materials Science Anhui Normal University Wuhu P. R. China; ^4^ Jinan Guoke Medical Technology Development Co., Ltd Shandong P. R. China; ^5^ Tianjin Union Medical Center The First Affiliated Hospital of Nankai University Tianjin P. R. China

**Keywords:** AI information encryption, persistent luminescence, phosphorescence quantum yield, silicon nanodots, ultralong lifetime

## Abstract

Persistent luminescence materials typically encounter an intrinsic trade‐off between high phosphorescence quantum yield (PhQY) and ultralong phosphorescence lifetime. To overcome this limitation, we propose a strategy that immobilizes silicon nanodots (SiNDs) within a dual‐functional composite matrix. The SiNDs efficiently generate abundant triplet excitons through intersystem crossing processes and simultaneously exhibit high PhQYs. Importantly, the urea‐paraformaldehyde‐derived matrix provides both the spatial confinement of molten urea and the extensive hydrogen‐bonding network of the urea‐formaldehyde resin. This synergistic configuration effectively immobilizes triplet excitons and suppresses nonradiative decay pathways. As a result, the material exhibits a remarkable PhQY of 81.04% together with an ultralong afterglow lifetime of 3.44 s. Furthermore, the energy transfer strategy further extends the persistent afterglow into the deep‐red region (702 nm). Leveraging the tunable afterglow colors and time‐resolved luminescent characteristics, an artificial intelligence‐assisted information encryption system was successfully developed. This work demonstrates that integrating SiNDs with a dual‐characteristic matrix provides a promising approach to concurrently achieving high PhQYs and ultralong lifetimes, thereby broadening the application scope of ultralong‐afterglow materials and guiding the rational design of next‐generation persistent luminescence materials.

## Graphical abstract

Schematic illustration of SiNDs composite materials synthesis and its internal photophysical process mechanism. And an AI‐assisted dynamic information encryption process.

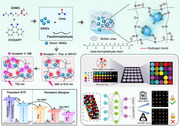

## 1 Introduction

Persistent luminescence materials, which exhibit photoluminescence‐based light storage and release properties, have garnered significant attention from both academic and engineering communities for their widespread applications in sensing, information security, optical imaging, and biotherapeutics [1, 2, 3, 4, 5]. This persistent luminescence mode, termed persistent afterglow, encompasses persistent room‐temperature phosphorescence (RTP) and thermally activated delayed fluorescence (TADF) [6, 7, 8]. Nevertheless, the intrinsic trade‐off between phosphorescence quantum yield (PhQY) and phosphorescence lifetime (τ*_p_*) presents a significant challenge, as most current persistent luminescence materials struggle to achieve high PhQY and ultralong lifetime simultaneously [9, 10]. For example, conventional rare‐earth‐based inorganic luminescent materials or metal‐organic frameworks can achieve high PhQY through enhanced molecular rigidity and heavy‐atom effects, but the corresponding photoluminescence lifetimes typically fall within the nanosecond (ns) to millisecond (ms) range, and these systems often present challenges such as high costs, demanding synthesis conditions, and potential toxicity [11, 12, 13]. Conversely, pure organic systems based on small organic molecules can achieve long τ*_p_*, but they necessitate complex synthetic routes to obtain specific molecular structures and cumbersome purification processes [14, 15, 16]. Consequently, the development of cost‐effective fabrication methods for ultralong persistent luminescence materials that simultaneously achieve high PhQYs and ultralong lifetimes remains a significant challenge.

Silicon nanodots (SiNDs) are prominent photofunctional materials in optoelectronics and biomedicine, distinguished by a stable electronic configuration that combines nanomaterial plasticity with specific photophysical characteristics [17, 18]. Previous work has reported that SiNDs can augment the photoluminescence quantum yield (PLQY) of materials. This effect is attributed to the electron‐donating nature of silane functional groups within SiNDs, which elevates local electron density and promotes efficient electron‐hole radiative recombination [19, 20, 21]. Notably, our previous research has demonstrated that Si─C and Si─O covalent bonds inside SiNDs stabilize triplet excitons and inhibit nonradiative transition processes, thereby extending their afterglow lifetime [22]. However, most SiNDs‐related phosphorescent materials currently exhibit phosphorescence lifetimes that fall short of the ultralong standard (>1.0 s), which significantly impedes the advancement of SiNDs‐based persistent luminescence materials [23, 24, 25, 26, 27, 28]. Consequently, devising effective strategies to leverage SiNDs' properties for achieving materials with both high phosphorescence quantum yields and ultralong phosphorescence lifetimes is paramount for developing high‐performance phosphorescent systems.

Rigid matrix‐assisted strategies have been widely employed to achieve efficient phosphorescence by restricting intramolecular motion and shielding emitters from atmospheric quenchers [29, 30, 31, 32]. However, most conventional matrices are composed of single components, which rarely integrate strong rigidity, dense hydrogen bonding sites, and excellent oxygen barrier capabilities within one system. Such limitations inevitably lead to enhanced nonradiative decay and diminished phosphorescence performance. For instance, the polyvinyl alcohol (PVA) matrices exhibit insufficient rigidity, and their abundant hydrophilic groups readily absorb moisture and oxygen from the environment, thereby quenching the triplet excitons of guest phosphorescent molecules [33, 34, 35]. Conversely, mesoporous frameworks offer effective confinement but lack abundant hydrogen bonding interactions necessary for exciton stabilization [36, 37]. Therefore, the rational design of advanced phosphorescent centers coupled with multifunctional matrix architectures capable of simultaneously providing rigidity, hydrogen bonding, and oxygen isolation is crucial for the development of next‐generation persistent luminescent materials.

**SCHEME 1:**
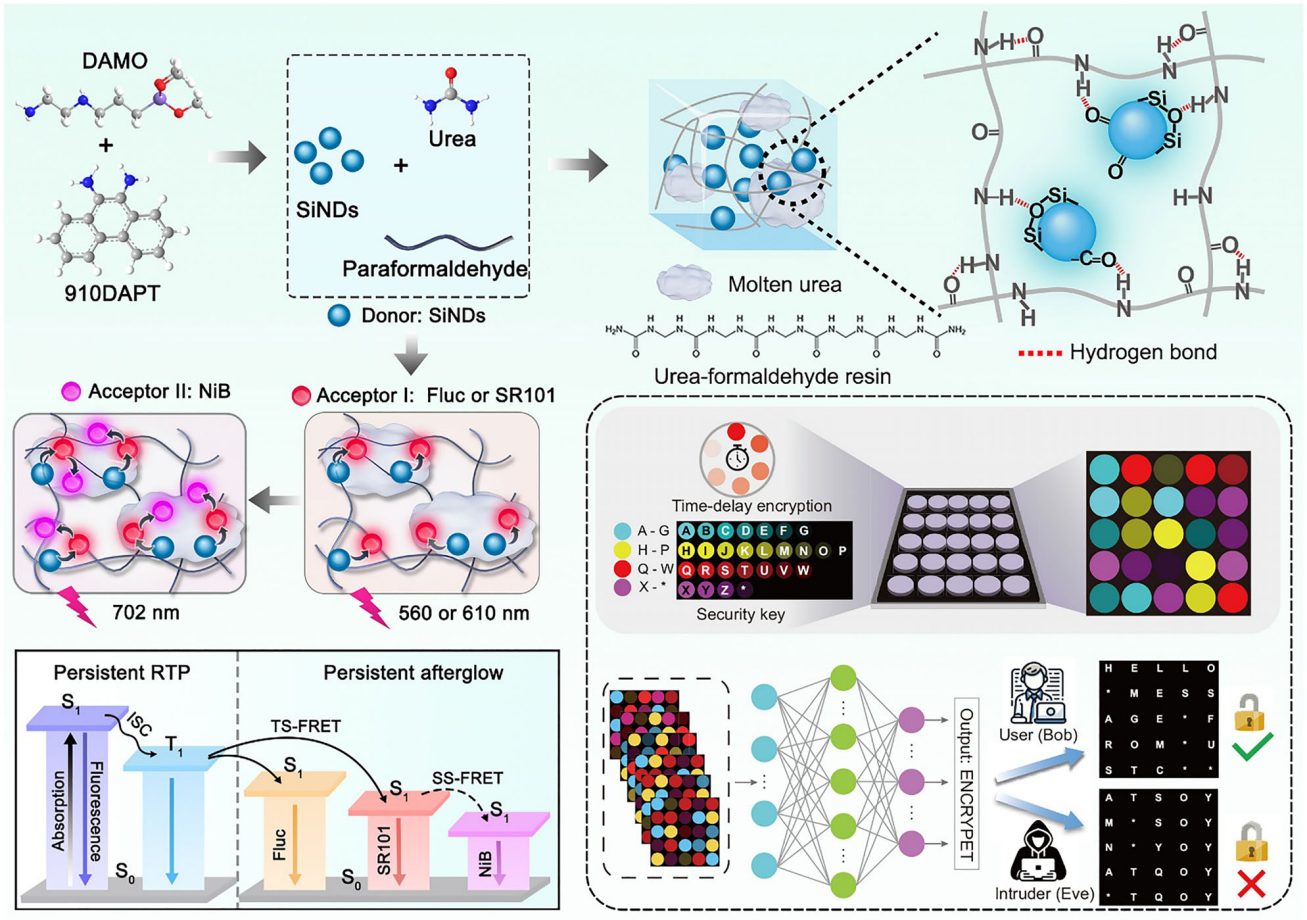
Schematic illustration of SiNDs composite materials synthesis and its internal photophysical process mechanism. And AI‐assisted dynamic information encryption process.

To address these limitations, we developed a facile hydrothermal strategy to synthesize SiNDs as phosphorescent centers, followed by their immobilization within a rigid matrix formed through the in situ reaction of urea and paraformaldehyde (Scheme 1). This approach yielded persistent luminescence composites exhibiting both high PhQYs and ultralong afterglow lifetimes. During the matrix formation, part of the urea underwent melting and recrystallization, while another fraction reacted with paraformaldehyde to generate a urea‐formaldehyde resin network. The resulting composite matrix comprising the molten urea domains and urea‐formaldehyde resin framework effectively restricted nonradiative relaxation pathways and stabilized triplet excitons within the SiNDs. Upon UV excitation, the SiNDs@U+F composites displayed remarkable cyan persistent phosphorescence lasting up to 52 s, with a measured lifetime of 3.44 s. Moreover, through an energy transfer mechanism between SiNDs and commercial organic dyes embedded in the matrix, multicolor afterglow emissions were successfully realized. Capitalizing on their ultralong and color‐tunable luminescence, SiND‐based composites were further employed to design dynamic information encryption systems, demonstrating promising potential in advanced optoelectronic and anti‐counterfeiting applications.

## 2 Results and Discussion

### 2.1 Preparation and Characterization of the SiNDs@U+F Composite

**FIGURE 1:**
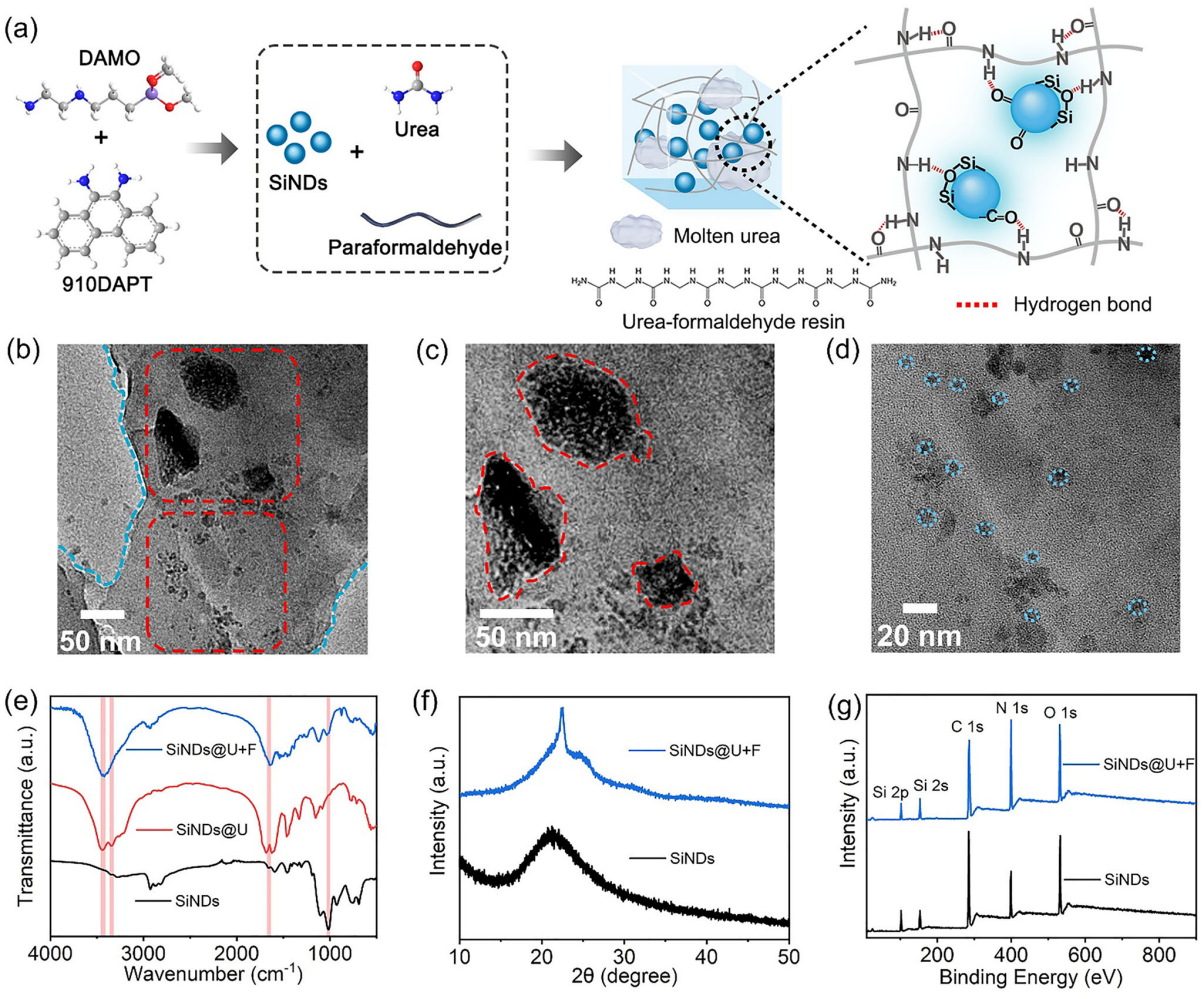
(a) Schematic diagram of the synthesis process and structure of SiNDs@U+F. (b) TEM image of SiNDs@U+F. Images of SiNDs immobilized in (c) molten urea and (d) urea‐formaldehyde resin structures. (e) XRD, (f) FT‐IR, and (g) XPS images of the SiNDs and SiNDs@U+F.

SiNDs were synthesized by a simple one‐step hydrothermal method using N‐[3‐(Trimethoxysilyl) propyl] ethylenediamine (DAMO) and 9,10‐diaminophenanthrene (910DAPT) as precursors. As shown in Figure S1, SiNDs are uniform in size, with an average particle size of 5.25 nm. After purification and lyophilization, the SiNDs were redissolved in ultrapure water and subsequently subjected to a second hydrothermal reaction with urea and paraformaldehyde, yielding the composite material designated SiNDs@U+F. We speculate that during the formation of the composite matrix, a portion of urea underwent melting and recrystallization to generate molten urea domains [38] while another fraction reacted with paraformaldehyde through a condensation process, producing linear urea‐formaldehyde resin polymer chains. The presence of abundant C = O and N‐H functionalities enables these polymer chains to bend and intertwine through electrostatic and hydrogen‐bonding interactions, ultimately assembling into chain‐like clustered structures [39]. These chain‐like clusters, in conjunction with the molten urea domains, form a robust hybrid matrix through extensive hydrogen bonding, within which the SiNDs are uniformly and rigidly immobilized (Figure 1a). This structure can be corroborated by the transmission electron microscopy (TEM) image of the materials (Figure 1b–d), in which the molten urea and the urea‐formaldehyde resin structure are in the background, and the SiNDs are immobilized in the matrix. The transmission electron microscopy‐energy dispersive spectroscopy (TEM‐EDS) images of SiNDs@U+F show that all four elements (C, N, O, Si) are distributed in the material (Figure S2), further confirming the doping of SiNDs into the composite matrix [40]. In addition, the TEM image of the material obtained by combining SiNDs and commercial urea‐formaldehyde resin, which we named SiNDs@UF, shows that the SiNDs are immobilized in the matrix formed by the urea‐formaldehyde resin without the appearance of molten urea (Figure S3). This finding indicates that a portion of the urea underwent a melting recrystallization process during the hydrothermal reaction.

To further clarify the structural composition in the composite matrix, three reference samples were prepared following the same conditions described in the Supporting Information (SI): hydrothermally treated urea (denoted as e@U), hydrothermally synthesized urea‐formaldehyde resin (e@UF), and hydrothermally treated urea and paraformaldehyde mixture (e@U+F). Differential scanning calorimetry (DSC) measurements (Figure S4a) show that the melting point of urea is 135°C [41]. Thermogravimetric analysis (TGA) indicates that urea does not undergo significant weight loss at 150°C, the temperature employed in the hydrothermal synthesis, suggesting that urea undergoes a melting recrystallization process during the formation of the composite matrix. The Fourier transform infrared (FT‐IR) spectra of these samples are shown in Figure S4b. A characteristic band at 1464 cm^−1^, assigned to the antisymmetric C‐N stretching vibration associated with urea‐urea interactions [42], is clearly observed in e@U and e@U+F but is absent in e@UF. In addition, the peak near 3347 cm^−1^ in e@U (attributed to ‐NH_2_ groups), and the band at approximately 3326 cm^−1^ in e@UF (associated with N‐H moieties in the linear resin polymers) are both absent [38, 43]. In contrast, e@U+F exhibits an enhanced band at 3438 cm^−1^, corresponding to secondary amine stretching vibrations, indicating the occurrence of condensation reactions between urea and formaldehyde [39].

Raman spectroscopy, X‐ray diffraction (XRD), and nuclear magnetic resonance (NMR) analyses were further employed to compare the three materials. As shown in Figure S4c, Raman bands at 960 cm^−1^ attributed to C─N stretching, and 1011 cm^−1^ attributed to NCN symmetric stretching mode are present in e@U and e@U+F but absent in e@UF, indicating that urea‐related structural motifs persist in the composite matrix [44, 45]. XRD patterns (Figure S4d) show a diffraction feature at 22.18° in all three samples; e@U+F and e@UF are found to have similar characteristic peaks, indicating that the product of the polycondensation reaction between urea and paraformaldehyde maintains the basic structure of urea‐formaldehyde resin. In the ^1^H NMR spectra (Figure S4e, f), e@UF exhibits characteristic resonances in the range of 6.63–6.94 ppm, which are also observed in e@U+F. Notably, e@U+F additionally shows a resonance at 5.55 ppm, which can be attributed to urea‐related protons [46]. These results collectively support that the hybrid matrix consists of both urea‐formaldehyde resin and molten urea components.

The presence of SiNDs within the composite material and their interaction mechanisms with the matrix were investigated using the same characterization approach. The material obtained from the hydrothermal treatment of SiNDs and urea is denoted as SiNDs@U in SI. In the Raman spectrum of SiNDs@U (Figure S5), a characteristic band located at 3470 cm^−1^ is observed, which can be assigned to the N‐H stretching vibration [47]. Upon the introduction of paraformaldehyde, this band redshifts to 3354 cm^−1^ in SiNDs@U+F. This pronounced redshift is attributed to changes in the local chemical environment of the N─H groups [47]. The incorporation of paraformaldehyde promotes the generation of clustered urea‐formaldehyde resin structures, which leads to a denser hydrogen‐bonding network within the matrix [39]. The formation of hydrogen bonds weakens the N─H bond strength, resulting in a decrease in the corresponding vibrational wavenumber [47]. To further corroborate this interpretation, the FT‐IR spectra of SiNDs, SiNDs@U, and SiNDs@U+F were compared, as shown in Figure 1e. For pristine SiNDs, the bands at 1648 cm^−1^ and 1017 cm^−1^ can be assigned to the stretching vibration of C═O/C═N groups and the asymmetric stretching vibration of Si─O─Si, respectively [48, 49, 50]. After incorporation into the composite matrix, these bands redshift to 1643 and 1008 cm^−1^ in SiNDs@U+F, indicating interactions between these functional groups and the surrounding matrix. And notably, the C═O/C═N bond has strong spin‐orbit coupling properties, which can efficiently populate triplet excitons through the intersystem crossing (ISC) process, which is a main source of phosphorescence [50]. In addition, in the FT‐IR spectrum of SiNDs@U, the N─H stretching band is located at 3448 cm^−1^ redshifts to 3440 cm^−1^ in SiNDs@U+F, which is in good agreement with the Raman results. It is well established that hydrogen bonding interactions arise when electronegative oxygen atoms withdraw electron density from the hydrogen atoms of ‐NH‐/‐NH_2_‐ groups, typically resulting in a redshift of the associated stretching vibrations [51, 52]. Therefore, the concurrent redshifts observed in both Raman and FT‐IR spectra provide strong spectroscopic evidence for the formation of hydrogen bonds between N‐H groups in the matrix and C═O and Si─O─Si functionalities [48, 53]. These interactions indicate that SiNDs interacts with urea‐formaldehyde resin through hydrogen bonds, and that the incorporation of paraformaldehyde leads to a more abundant and complex hydrogen‐bonding network compared with the urea‐derived matrix alone.

The XRD characterization results (Figure 1f) show that the broad amorphous peak at 21.21° corresponds to the broad amorphous peak of the SiNDs, while the sharper diffraction peaks in the composite indicate higher internal crystallinity of the material [54]. The internal composition of the material was further analyzed via X‐ray photoelectron spectroscopy (XPS), and Figure 1g shows that both the SiNDs and SiNDs@U+F material contain four elements: C, N, O, and Si. The full XPS spectra of SiNDs@U+F display peaks corresponding to carbon (41.20 %), nitrogen (27.74 %), oxygen (22.79 %), and silicon (8.27 %); these characteristic peaks are observed at 286.1, 399.1, 531.1, 153.1, and 102.1 eV, respectively. The C1s spectrum (Figure S6) exhibited three peaks at 284.4, 285.8, and 288.5 eV, which are assigned to C─Si, C─N, and C═O moieties, respectively. The N1s XPS spectrum was fitted to three components: N‐Si (398.8 eV), amino N (399.5 eV), and graphitic N (400.3 eV) (Figure S6). The O1s spectrum (Figure S6) was deconvoluted into three peaks at 531.2, 532.4, and 532.9 eV assigned to C═O, O─Si, and C─O bonds, respectively. The Si 2p spectrum is composed of three peaks corresponding to Si─C (101.4 eV), Si─N (102.2 eV), and Si─O (102.8 eV) (Figure S6). The high‐resolution XPS spectra and fitting results of SiNDs are shown in Figure S7 and Table S1. The above characterization results show that the SiNDs are uniformly dispersed in the composite matrix (molten urea and urea‐formaldehyde resin) and that the luminescent centers of the SiNDs can be stabilized in the matrix through space constraints and hydrogen bonding.

### 2.2 Photoluminescence Performance and Mechanism of SiNDs@U+F Composite

**FIGURE 2:**
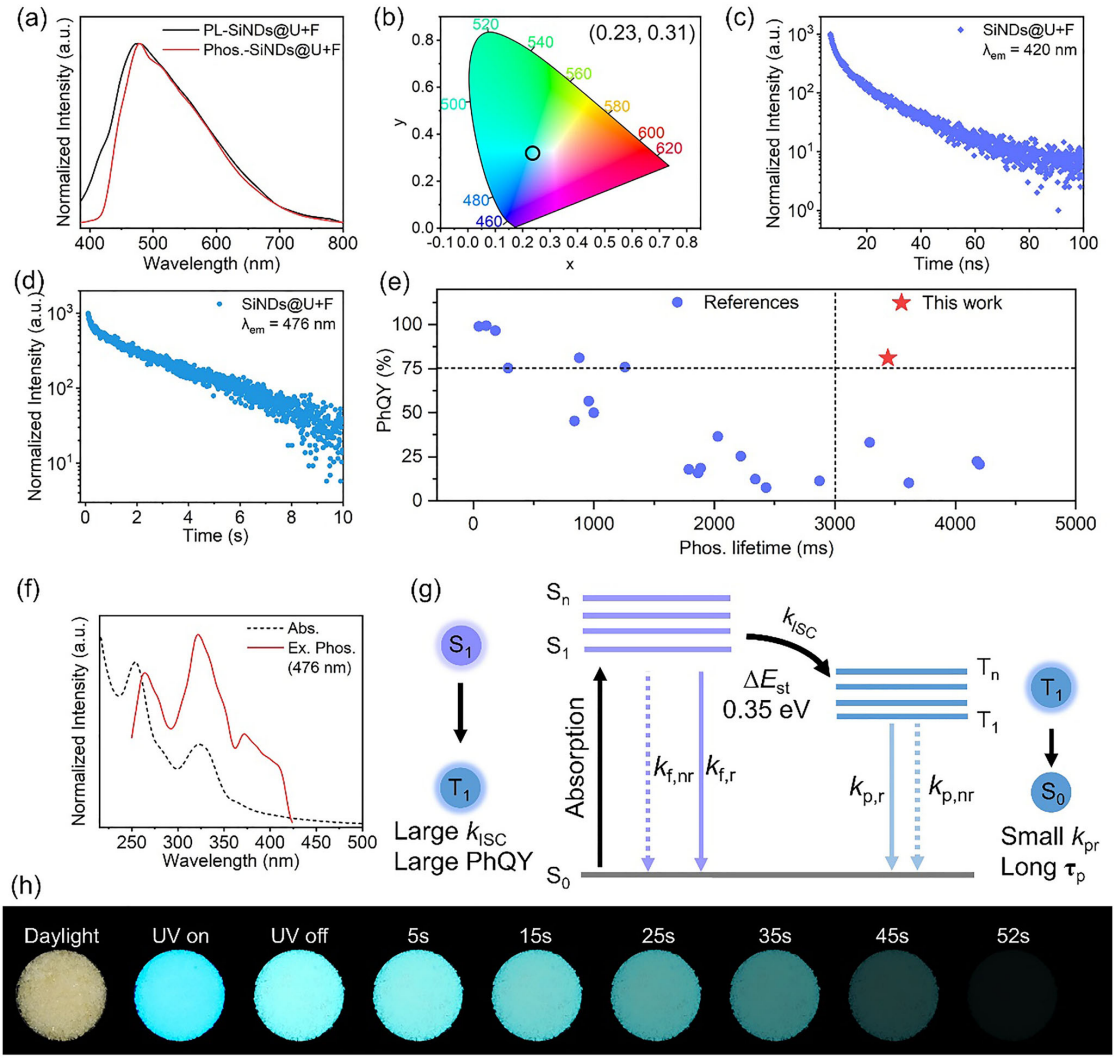
(a) Steady‐state photoluminescence and phosphorescence spectra of SiNDs@U+F under 365 nm excitation. (b) CIE plot coordinates of SiNDs@U+F. (c) Time‐resolved phosphorescence and (d) fluorescence decay curves of SiNDs@U+F under 365 nm excitation. (e) Comparison of PhQY RTP and phosphorescence lifetimes between this work and the previously reported materials. (f) UV absorption spectrum of SiNDs@U+F, and excitation spectrum when phosphorescence emission is 476 nm. (g) The Jablonski diagram and theoretical calculations of SiNDs@U+F, symbols meanings and values are included in Table S3.(h) Physical maps of SiNDs@U+F under daylight, 365 nm UV lamp on and off with time.

The photophysical properties of SiNDs@U+F were explored. As shown in Figure 2a, the steady‐state and delayed photoluminescence spectra of SiNDs@U+F reveal blue emission at 476 nm (*λ*_ex_ = 365 nm), which corresponds to the phosphorescence emission of the material. The emission band near 420 nm corresponds to fluorescence emission. By calculating the Commission Internationale de l'Éclairage (CIE) coordinates (Figure 2b), the phosphorescence coordinates of the SiNDs@U+F are found to be (0.23, 0.31). Figure 2h shows the physical pictures of SiNDs@U+F in different environments; this material is a yellowish powder in daylight, emits blue light under 365 nm excitation, and emits cyan phosphorescence after the excitation is turned off, which lasts for 52 s under naked eye observation. The absolute PLQY of the material is 91.99%, and its PhQY is calculated to be 81.04% (Figure S8). Its phosphorescence lifetime at 476 nm is 3.44 s (Figure 2c), and its fluorescence lifetime is 12.21 ns (Figure 2d). Compared with previously reported materials (Figure 2e, Table S2), this material is currently one of the best‐performing persistent luminescent materials, which has an ultralong phosphorescence lifetime (>3.0 s) and a high PhQY (>75.00%).

We explored the reasons for the excellent performance of the material in terms of both the luminescent center and the matrix structure. The UV‐visible absorption spectrum of SiNDs@U+F shows two absorption peaks at 254 nm and an absorption peak at 323 nm (Figure 2f), which are attributed to the π–π* transitions of the C═C bond and the n‐π* transitions of the C═O/C═N bond in SiNDs, respectively. Notably, the phosphorescence excitation and UV‐vis absorption spectra of the SiNDs@U+F have spectral overlap at 292–362 nm, suggesting that the C═O/C═N bonding of SiNDs@U+F is a reason for phosphorescence production [50]. To further investigate the luminescence process of SiNDs@U+F, we only chose 910DAPT as the precursor to obtain silicon‐free carbon nanodots (CDs) via hydrothermal reaction, and CDs@U+F were obtained by the same preparation process. Figure S9b shows a spectral overlap between the phosphorescence excitation and UV–vis absorption spectra of CDs@U+F at 300–363 nm, further suggesting that the phosphorescence emission is related to the C═O/C═N bonds in its structure. This is because the lone electron pairs of N and O have strong spin‐orbit coupling characteristics, which can promote the intersystem crossing process [55]. Additionally, the imidazole derivatives produced by 910DAPT during the hydrothermal process possess low energy polarization, which is favorable for enhancing spin‐orbit coupling, thus improving the ISC efficiency and increasing the number of triplet excitons [39]. The phosphorescence phenomenon of the two materials indicates that C═O/C═N and imidazole derivatives are the main factors contributing to the generation of RTP. However, CDs@U+F have a phosphorescence lifetime of 1.68 s and a PLQY of 56.93%, both of which are lower than those of SiNDs@U+F (Figure S9c, d). This difference arises because the unique Si‐C and Si‐O covalent bonding of the SiNDs has a fixing effect on the triplet excitons, which can prolong the phosphorescence lifetime. In addition, the *ΔE_ST_* of SiNDs@U+F (0.35 eV, Table S3) is lower than that of CDs@U+F (0.47 eV). According to the Franck‐Condon principle [56], a smaller value of *ΔE_ST_* results in a faster ISC rate (*k*_isc_ = 7.29 × 10^7^/s) and more efficient ISC. Coupled with silane's ability to improve the efficiency of electron‐hole radiation complexes by increasing the electron density, resulting in a higher PhQY of SiNDs@U+F.

To evaluate the general applicability of the proposed synthesis methodology for producing high PLQY and long‐lifetime phosphorescent materials, three types of SiNDs were synthesized using three different precursors. As shown in Table S4 and Figure S10, all three samples exhibit blue long‐lived phosphorescence under identical conditions. This suggests that the strategy provides a viable and generalizable approach for the development of high‐performance afterglow materials based on SiNDs.

To further evaluate the importance and effectiveness of the composite matrix for the triplet emission of SiNDs@U+F, we compared the phosphorescence wavelength and lifetimes of SiNDs@UF and SiNDs@U. The phosphorescence emissions of both materials are consistent with those of SiNDs@U+F (476 nm), but their phosphorescence lifetimes (1.24 s, 2.48 s) are lower than that of SiNDs@U+F (Figure S11a, b). The correlation between the phosphorescence lifetime and radiation rate indicates that the nonradiative transition losses within a single matrix are greater than those within the composite matrix. Owing to the combination of the rigidity of molten urea and the abundant hydrogen bonds within urea‐formaldehyde resin, the composite matrix can play a dual role in synergistically suppressing the vibrational dissipation of long‐lived triplet excitons. As shown in Figure 2g, the *k*_p,r_ of SiNDs@U+F is as low as 0.26, indicating that the composite matrix effectively suppresses energy loss caused by nonradiative transitions and triplet exciton quenching.

Additionally, we conducted further comparative studies on the photostability of the three materials. As shown in Figure S12a–c, all three materials exhibit negligible phosphorescence intensity decay under nitrogen and ambient air, indicating good air stability. In aqueous environments, SiNDs@U+F demonstrates significantly improved water resistance compared with SiNDs@UF and SiNDs@U, retaining more than 93% of its initial phosphorescence intensity over the tested duration. Figure S12d shows the phosphorescence intensity changes of the three materials in various oxidizing agents and organic solvents. A slight decrease in phosphorescence intensity is observed for all samples in oxidizing environments, which can be attributed to quenching of triplet excitons by dissolved oxygen [57]. In comparison, the materials remain largely stable in organic solvents, exhibiting minimal changes in emission intensity. Overall, these results indicate that all three materials possess good environmental stability, while SiNDs@U+F consistently shows superior resistance to water and oxidative conditions compared with SiNDs@UF and SiNDs@U. This enhanced stability can be attributed to the rigidity of molten urea within the abundant hydrogen bonding in the urea‐formaldehyde resin, which more effectively stabilizes triplet excitons, suppresses nonradiative decay pathways, and isolates the emissive centers from external quenchers such as water and oxygen, relative to single‐component matrices.

Based on the combined structural characterization and photophysical analyses, the luminescence mechanism of the SiNDs@U+F can be rationalized as follows (Figure 1a): (i) Luminescence center formation: SiNDs serve as the luminescence center, where the incorporation of silicon enhances both the PLQY and exciton population. Facilitated by the efficient ISC mediated through C═N/C═O functionalities and imidazole‐derived moieties, a high triplet exciton population is generated, contributing to the elevated PhQY. (ii) Matrix confinement by molten urea: A portion of urea undergoes melting and recrystallization to form a highly crystalline and rigid domain, which restricts molecular vibrations and rotations of the chromophoric units, thereby reducing nonradiative relaxation. (iii) Stabilization through urea‐formaldehyde network: Concurrently, the polycondensation of urea and paraformaldehyde produces urea‐formaldehyde resin chain clusters rich in hydrogen‐bonding interactions. These clusters effectively immobilize SiNDs and stabilize triplet excitons by providing an extensive hydrogen‐bonded framework that further suppresses nonradiative transitions. The coexistence of the crystalline molten‐urea domains and hydrogen‐bonded resin networks thus establishes a dual‐confinement matrix that efficiently prevents triplet‐state quenching and prolongs the phosphorescence lifetime. Additionally, the rigid framework isolates SiNDs from external quenching species such as moisture and oxygen, ensuring long‐term luminescence stability. Collectively, these results demonstrate that immobilizing SiNDs within a dual‐characteristic composite matrix represents a viable strategy for achieving both high PhQYs and ultralong persistent afterglow.

### 2.3 Regulation of Multi‐Color and Long‐Lifetime Afterglow Materials

**FIGURE 3:**
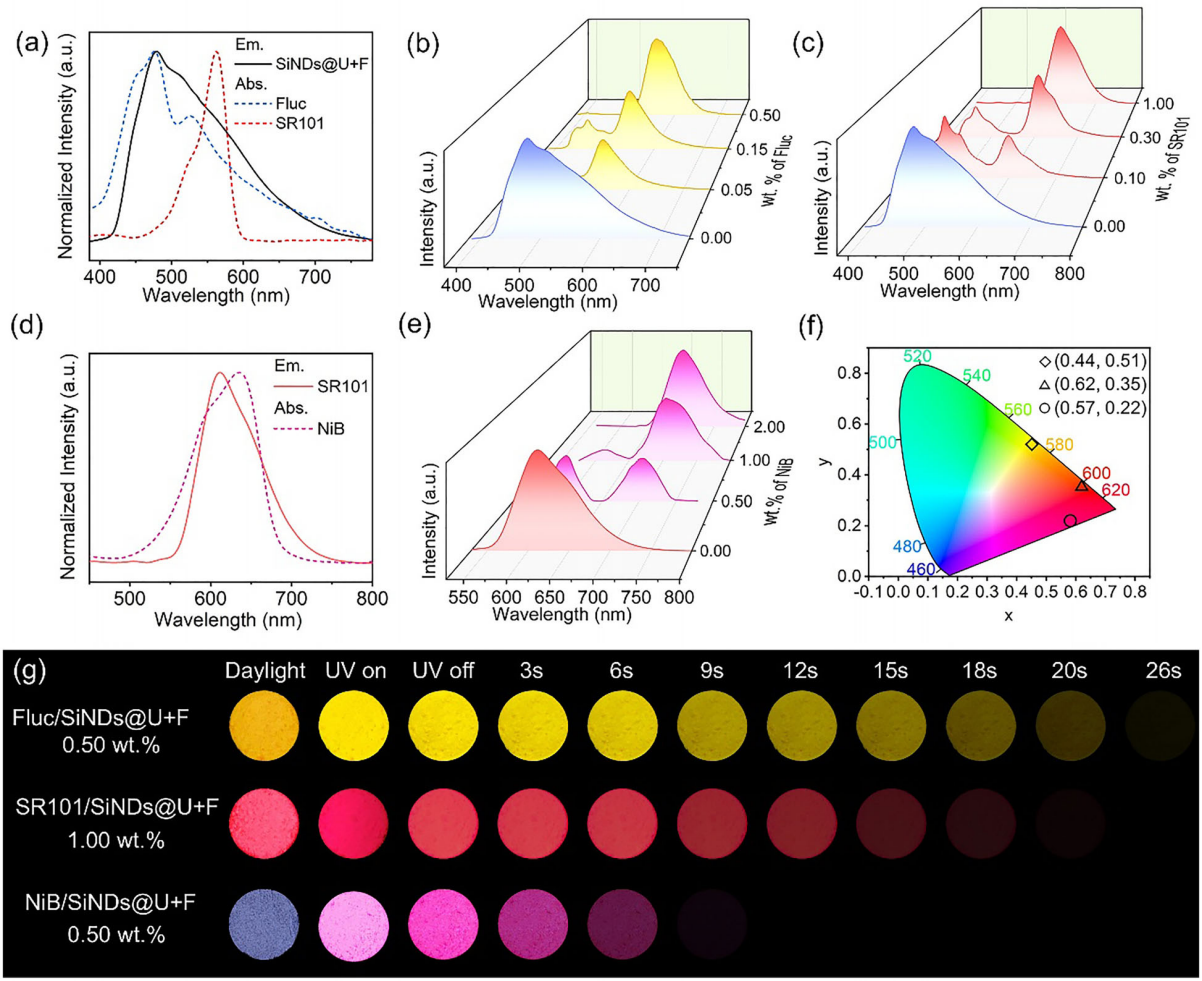
(a) Absorption spectra of the guests (Fluc, SR101) and phosphorescence spectrum of SiNDs@U+F. (b) Afterglow emission spectra of Fluc/SiNDs@U+F at different Fluc doping weight concentrations. (c) Afterglow emission spectra of SR101/SiNDs@U+F at different SR101 doping weight concentrations. (d) Absorption spectrum of the guests (NiB) and afterglow spectrum of Fluc/SiNDs@U+F. (e) Afterglow emission spectra of NiB/SiNDs@U+F at different NiB doping weight concentrations. (f) CIE 1931 coordinates of three systems (Fluc/SiNDs@U+F, SR101/SiNDs@U+F and NiB/SiNDs@U+F). (g) Photographs of three systems taken after the removal of the 365 nm excitation lamp.

**FIGURE 4:**
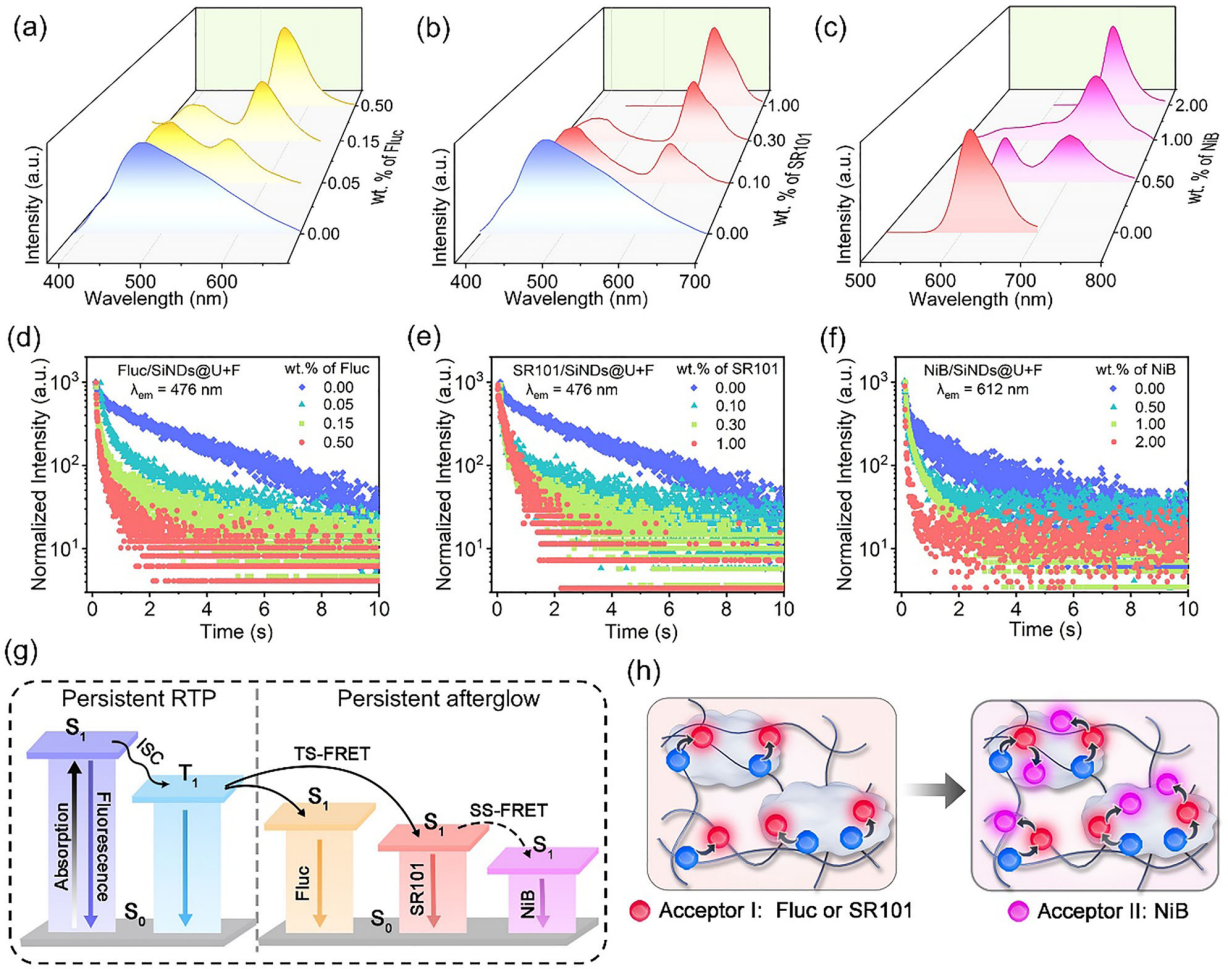
(a) Fluorescence spectra of Fluc/SiNDs@U+F at different Fluc doping weight concentrations. (b) Fluorescence spectra of SR101/SiNDs@U+F at different SR101 doping weight concentrations. (c) Fluorescence spectra of NiB/SiNDs@U+F at different NiB doping weight concentrations. (d–f) Time‐resolved afterglow decay curves of three systems with gradually increasing acceptor doping weight concentration; (g) Mechanism of TS‐FRET and SS‐FRET processes. (h) Schematic diagram of the internal structure of the three materials.

Based on the ultralong phosphorescence lifetime and high PhQY of SiNDs, as well as the immobilization of triplet excitons by the composite matrix, we modulate the persistent luminescence spectra of the composites according to the Förster resonance energy transfer (FRET) principle [58]. The occurrence of energy transfer requires effective spectral overlap between the emission spectrum of the energy donor and the absorption spectrum of the energy acceptor [59]. Therefore, we initially used the commercial dye fluorescein (Fluc) and sulforhodamine 101 (SR101) as energy acceptors and named these two systems Fluc/SiNDs@U+F and SR101/SiNDs@U+F, respectively. The absorption spectrum of Fluc (Figure 3a) ranges from 400–700 nm, the absorption spectrum of SR101 ranges from 470–600 nm, and the absorption spectra of both materials overlap well with the phosphorescence spectrum of the SiNDs@U+F. As shown in Figure 3b, with the gradual increase in the Fluc doping weight concentration from 0.00 to 0.50 weight% (wt.%), the afterglow intensity of the energy donor decreases, whereas that of the energy acceptor increases. The change in the ratio between the two afterglow emission bands indicates a triplet‐singlet FRET (TS‐FRET) process between the energy donor and the acceptor [60], and the afterglow peak stabilizes at 560 nm when the concentration of Fluc reaches 0.50 wt.%. The corresponding afterglow lifetime is 2.51 s (Figure S11c). Moreover, Figure 4a shows the change in the ratio between the two steady‐state photoluminescence spectra within the Fluc/SiNDs@U+F system, which indicates that the fluorescence also involves energy transfer. Ultimately, the fluorescence peak stabilizes at 560 nm when the concentration of Fluc/SiNDs@U+F reaches 0.50 wt.%, and the corresponding fluorescence lifetime is 7.96 ns (Figure S11b), with the PLQY reaching 92.46% (Figure S11d).

This phenomenon was also observed in the SR101/SiNDs@U+F system (Figures 3c and 4b), in which the steady‐state photoluminescence and afterglow intensities of the energy acceptors in the SR101/SiNDs@U+F system gradually increase as the SR101 doping concentration increases from 0.00–1.00 wt.%, and the final fluorescence and afterglow emission peaks of the SR101/SiNDs@U+F system stabilize at 612 nm when the SR101 concentration is 1.00 wt.%. The corresponding fluorescence and afterglow lifetimes are 4.36 ns and 2.36 s, respectively (Figure S12b, c), and the PLQY reaches 95.50% (Figure S12d). Notably, the PLQY of both materials is higher than that of SiNDs@U+F. We first reacted the fluorescent molecules with urea and paraformaldehyde, and then analyzed the structural changes via XRD. Fluc@U+F and SR101@U+F exhibit high crystallinity, whereas the diffraction peaks of the material containing SiNDs become wider and the structure becomes more amorphous, suggesting potential conformational adjustments during the formation of the material (Figures S11e, and S14e). Subsequently, the fluorescence anisotropy of the dye molecules was analyzed, and the corresponding results are shown in Figure S15. As references, Fluc@U+F and SR101@U+F embedded in the polymer matrix without SiNDs exhibit very low anisotropy values (*r* = 0.003), indicating a high degree of rotational freedom and efficient depolarization of the excited‐state emission. In contrast, the anisotropy values increase to 0.013 for Fluc/SiNDs@U+F and 0.086 for SR101/SiNDs@U+F, respectively. This increase suggests that the rotational motion of the dye molecules is partially restricted in the presence of SiNDs, resulting in reduced depolarization and higher anisotropy values [61, 62]. Therefore, we speculate that during the co‐encapsulation of SiNDs and dyes into the matrix, repulsive forces between overlapping electron clouds induce steric effects between host‐guest molecules. These steric effects influence the spatial conformation of host‐guest molecules, leading to conformation adjustments that achieve perfect matching with the host cavity. This adaptive host‐guest process may be related to the hyperconjugation effect and suppresses nonradiative transition in the triplet state, significantly enhancing persistent luminescence efficiency [63].

Furthermore, based on the emission peak position of SR101/SiNDs@U+F, we choose Nile blue (NiB), whose absorption peak is located at 500–700 nm, as the energy acceptor of SR101/SiNDs@U+F (Figure 3d). With the gradual increase in the proportion of NiB, the steady‐state photoluminescence and afterglow emission peaks of the material transition from 612 to 702 nm, reaching deep red emission (Figures 3e and 4c). When the NiB concentration is 2.00 wt.%, the final fluorescence and afterglow emission peaks of the NiB/SiNDs@U+F system stabilize at 702 nm. The corresponding fluorescence and afterglow lifetimes are 5.73 ns and 1.08 s, respectively (Figure S13b, c), and the PLQY reaches 32.48% (Figure S13d). Figures S13f, S14f, and S16f show the physical diagrams of the three dyes at different doping weight concentrations, respectively, which show that the materials with different ratios have uniform colors. With an increasing amount of the dye molecules, the overall color of the material under daylight gradually approaches the characteristic color of the dye, where Fluc/SiNDs@U+F is a yellow powder (Figure 3 g), and the persistent afterglow after the UV excitation is turned off is yellow. The SR101/SiNDs@U+F and NiB/SiNDs@U+F are red and indigo powder, and the persistent afterglow after the UV excitation is turned off is red and claret, respectively. The afterglow colors of the three systems are consistent with the calculated afterglow CIE coordinates (Figure 3f).

The resonance energy transfer process was further explored by monitoring the variation in afterglow lifetimes (Figure 4d–f). The gradual decrease in the afterglow lifetime of the energy donors with increasing acceptor concentration clearly indicates the occurrence of an efficient energy‐transfer process within the system. Based on the above analysis results, the internal configuration of the multicolor afterglow composites can be rationalized as illustrated in Figure 4g–h. The fluorescent molecules are homogeneously incorporated into the matrix as energy acceptors. After cessation of excitation, the spectral overlap between the triplet emission of the SiNDs and the absorption band of the fluorescent dye facilitates the transfer of triplet exciton energy to the singlet excited state of the acceptor molecules, giving rise to the TS‐FRET process. In particular, a stepwise energy‐transfer mechanism is operative in the NiB system. Initially, triplet excitons from SiNDs transfer their energy to the intermediate dye SR101; subsequently, part of the singlet exciton energy in SR101 is relayed to the final acceptor NiB through a singlet‐singlet FRET (SS‐FRET) pathway [58]. This sequential process ensures efficient cascade energy transfer and enables fine control over the emission color, and the energy transfer efficiency results of the three materials are shown in Table S5. The optical characteristics of these multicolor afterglow composites confirm the feasibility of this energy‐transfer design, establishing a versatile approach for color‐ and time‐resolved phosphorescence modulation. Furthermore, this strategy provides valuable insights for extending afterglow emissions toward the near‐infrared region, thereby expanding the functional scope of persistent luminescent materials.

### 2.4 Applications of SiNDs@U+F Composite

In the traditional optical anticounterfeiting field, fluorescent labels mainly show a single luminescence pattern under UV or near‐infrared irradiation, which is easily counterfeited by substitutes with similar luminescence behavior. Continuous luminescent materials have multidimensional luminescent properties, including the luminescent color, intensity, and decay time, which can effectively improve the encryption level. The multiple persistent luminescent materials designed in this work possess unique time‐resolved emission properties, which are of great practical importance in advanced anticounterfeiting technologies. As shown in Figure S17, a flower pattern was designed on the basis of the four materials, and after UV excitation is turned off, the four materials exhibit very different colors, indicating that a simple encryption pattern can be achieved on the basis of the luminescent colors.

On the basis of the fluorescence and different durations of afterglow for various doping ratios in the Fluc/SiNDs@U+F system, a visual information encryption system with a luminescent state and binary code/Morse code was designed in combination with Morse code. In this system, the presence of a light signal is defined as “1” in binary code and “dot (.)” in Morse code. The absence of light is defined as “0” in binary code and “dot (.)” in Morse code. As shown in Figure S18, Fluc/SiNDs@U+F (0.50 wt.%, 0.05 wt.%) exhibits consistent yellow fluorescence when excited at 365 nm, representing erroneous information. After 26 s of de‐excitation, the Fluc/SiNDs@U+F (0.50 wt.%) afterglow signal disappears. Based on these rules, the authentic message is ultimately retrieved and identified as “silicon”.

With the advancement of artificial intelligence (AI) as a cutting‐edge tool, its applications in the biomedical field have garnered significant attention from scholars [64, 65, 66]. By integrating silicon nanomaterials with AI‐based anti‐counterfeiting models, we have designed a more adaptable data encryption model. As shown in Figure S19, the cryptosystem is designed to encrypt sequences of symbols drawn from a physical alphabet of 27 distinct afterglow markers. These were characterized by unique spectral and temporal decay profiles. For the cryptosystem, this alphabet is digitally represented by a color palette where each of the 27 unique colors is mapped to one of the 26 letters of the English alphabet and a placeholder symbol. This palette serves as the ground truth for the encryption and decryption tasks, where each symbol corresponds to a unique 27‐dimensional one‐hot vector.

The fundamental unit of plaintext for our system is a sequence, or row, of 5 such symbols. To prepare this data for the neural networks, each symbolic index is transformed into a 27‐dimensional one‐hot vector, where the element corresponding to the symbol's index is set to 1, and all other elements are 0. A single plaintext message, m, is therefore represented by the concatenation of five such one‐hot vectors, resulting in a 135‐dimensional vector that serves as the input to the encryption network. This one‐hot scheme provides a categorical representation of the data, which is well‐suited for the classification‐based loss function used in training.

**FIGURE 5:**
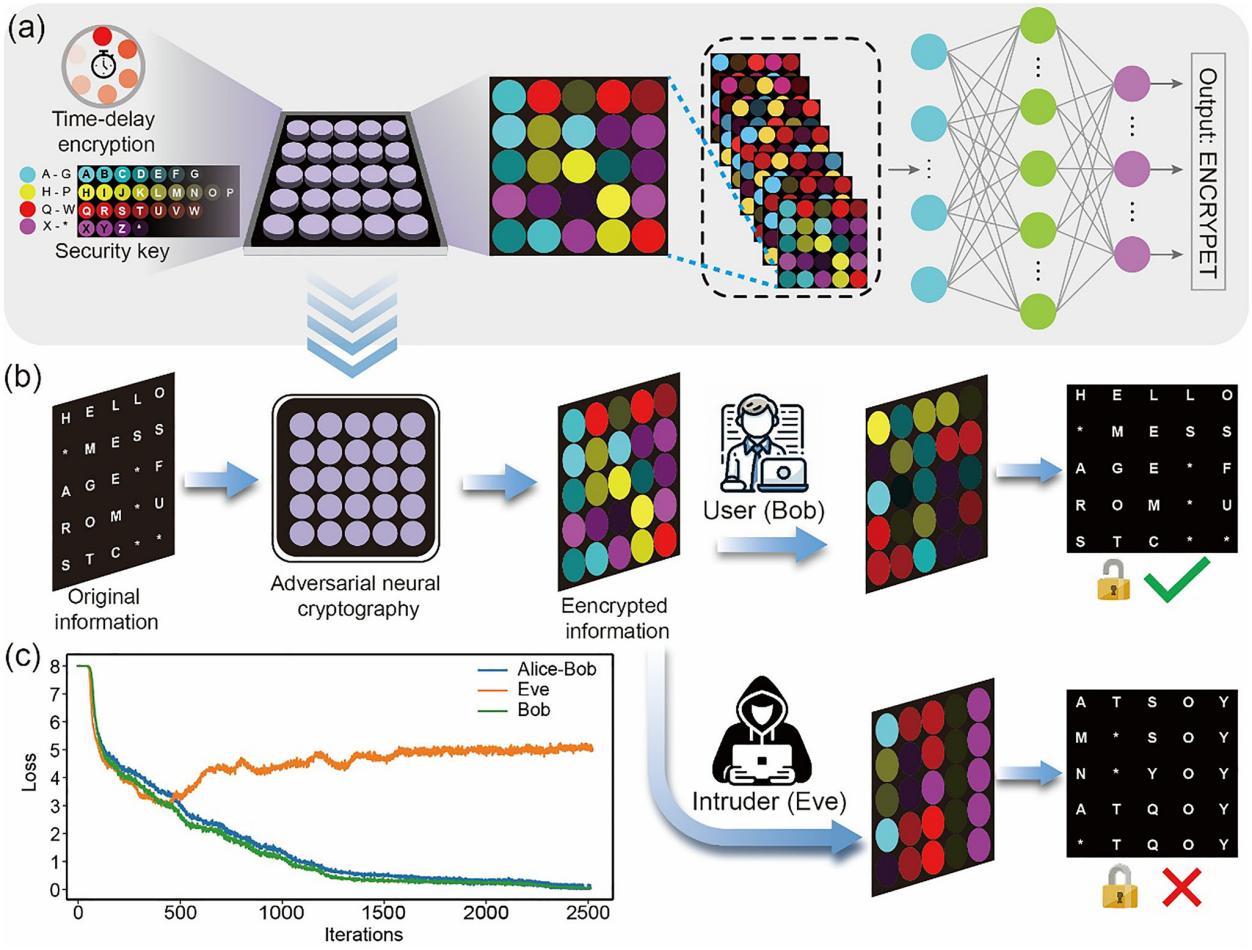
(a) The encryption system consists of a 5×5 information grid, where each position corresponds to a random symbol. All symbols undergo function processing to form the encrypted information. (b) The original information is encrypted by the encryption system and transmitted to the user, who uses the security key to decrypt the message correctly. Whereas an intruder without the key will get incorrect decrypted information. (c) Training dynamics of the adversarial neural cryptosystem.

We designed an end‐to‐end cryptographic system using an adversarial framework composed of three independent multi‐layer perceptrons (MLPs), designated Alice (encryptor), Bob (user), and Eve (intruder). All models were implemented in PyTorch. The networks share a similar architecture, consisting of a sequence of fully‐connected layers with LeakyReLU activations and dropout layers for regularization. Alice's network is engineered to encrypt a 135‐dimensional plaintext message, m, using a 64‐bit secret key, k, to produce a ciphertext, c. The plaintext and key are first concatenated before being passed through the network. A critical feature of Alice's design is its output layer, which uses the Gumbel‐Softmax function. This produces a differentiable, “soft” approximation of a one‐hot encoded vector for each of the 5 symbol positions (Figure 5a). This allows the network to make discrete, categorical choices for the ciphertext while maintaining a gradient for backpropagation. Bob's network is designed for decryption. It takes the ciphertext from Alice and the secret key, k, as input. These are flattened and concatenated, then processed by the MLP. Eve's network is architecturally similar to Bob's but is trained to decrypt the ciphertext without access to the key. Both Bob's and Eve's networks use a standard Softmax function in their output layers, producing a probability distribution over the 27 possible symbols for each of the 5 positions in the sequence.

Then, the system was optimized using an adversarial training strategy with cross‐entropy loss as the objective function, which is appropriate for the categorical nature of the task. The training was conducted in two phases. In the initial “warm‐up” phase, only the Alice and Bob networks were trained jointly. Their combined loss was solely Bob's reconstruction error, calculated as the cross‐entropy between Bob's predicted probability distributions and the original ground‐truth symbol indices. This phase continued until Bob's network could reliably decrypt Alice's messages. Once this criterion was met, the second phase began, introducing the adversarial network, Eve. In this phase, Alice and Bob's joint loss function was augmented with an adversarial term: *L_A, B_ = L_B_+max(0, M−L_E_)*, where *L_B_* is Bob's cross‐entropy loss, *L_E_* is Eve's cross‐entropy loss, and *M* is a predefined margin. This term penalizes Alice for creating ciphertexts that Eve can easily decrypt, forcing the system to learn a more secure encryption scheme. Concurrently, Eve was trained independently to minimize its own cross‐entropy loss, *L_E_*. The training regimen consisted of alternating optimization steps: two cycles for the Alice‐Bob system followed by one for Eve. Both optimization processes utilized the Adam optimizer and a batch size of 512.

The final information processing result is shown in Figure 5b. The original message, defined by a specific text phrase, is encrypted by Alice into a visually random pattern. This encrypted visualization is a pseudo‐representation, as the true ciphertext consists of probability distributions generated by the Gumbel‐Softmax function. Bob, using the secret key, perfectly reconstructs the original message, while Eve's attempt without the key results in a random, incorrect pattern. This explicitly shows the perfect recovery of the original letter sequence by Bob and the complete failure of Eve to extract meaningful information, confirming the establishment of a secure communication channel. Figure 5c shows the cross‐entropy loss for the Alice‐Bob system (blue), Bob alone (green), and Eve (orange) as a function of training iterations. The training process is phased: initially, only Alice and Bob are trained, and their loss curves converge rapidly. Eve's training commences only after Bob's loss falls below a predefined threshold, at which point its loss (orange) begins to decrease as it learns to attack the existing scheme. In response, the Alice‐Bob loss (blue) increases as it adapts its strategy to counter Eve. Ultimately, the Bob and Alice‐Bob losses converge toward zero, while Eve's loss is driven up and stabilizes at a high value, indicating that a secure and reliable communication channel has been established.

## 3 Conclusion

In summary, we have developed an economical, facile, and innovative strategy to integrate SiNDs into a composite matrix, effectively combining the high PLQY of SiNDs with the long‐afterglow enhancement capability of the matrix. The resulting materials exhibit an impressive PhQY of 81.04% and an ultralong afterglow lifetime of 3.44 s, accompanied by a visible cyan emission persisting for 52 s. These properties rank the material among the best‐performing persistent luminescent systems reported to date. The exceptional performance arises from the efficient ISC within the SiNDs and the effective suppression of triplet exciton dissipation by the surrounding matrix. The dual‐characteristic matrix offers distinct advantages over single‐component systems. Furthermore, through an energy transfer mechanism, incorporating commercial dyes enables precise modulation of both the emission color and afterglow duration, yielding multicolor SiND‐based composites. These materials not only demonstrate outstanding optical performance but also exhibit excellent potential in information encryption through AI models, highlighting their promising application in advanced anti‐counterfeiting technologies. Overall, this work provides valuable insights for the rational design of multicolor‐tunable phosphorescent materials with high PhQYs and ultralong lifetimes, paving the way toward their broader practical applications.

## Conflicts of Interest

The authors declare no conflict of interest.

## Supporting information




**Supporting File**: advs73811‐sup‐0001‐SuppMat.docx.

## Data Availability

The data that support the findings of this study are available in the supplementary material of this article.
