# Supplementary material for: Urea‐Formaldehyde Resin Confined Silicon Nanodots Composites: High‐Performance and Ultralong Persistent Luminescence for Dynamic AI Information Encryption
Source: Adv Sci (Weinh). 2026 Jan 20;13(17):e22820. doi: 10.1002/advs.202522820 (PMC13042435; doi:10.1002/advs.202522820)
Supplement: Supplementary file 1 — Supporting File: advs73811‐sup‐0001‐SuppMat.docx. [file ADVS-13-e22820-s001.docx]

***Supporting Information***

**Urea-Formaldehyde Resin Confined Silicon Nanodots Composites: High-Performance and Ultralong Persistent Luminescence for Dynamic AI Information Encryption**

*Yulu Liu ^a,d †^, Lei Cao ^a †^, Lele Gao ^a,c^, Panyong Wang ^a^, Qiannan You ^a^, Xinpei Pang ^a^, Li Li ^a,b^, Mingzheng Jia ^e^, Wen-Fei Dong ^a,b*^ and Minghui Zan ^a*^*

a. Department of Biomaterials and Stem Cells, Suzhou Institute of Biomedical Engineering and Technology, Chinese Academy of Science (CAS), Suzhou 215163, P. R. China

b. School of Biomedical Engineering (Suzhou), Division of Life Sciences and Medicine, University of Science and Technology of China, Hefei 230026, P. R. China

c. Anhui Key Laboratory of Biomedical Materials and Chemical Measurement, Key Laboratory of Functional Molecular Solids, Ministry of Education, College of Chemistry and Materials Science, Anhui Normal University, Wuhu 241002, P. R. China

d. Jinan Guoke Medical Technology Development Co., Ltd, Shandong, P. R. China

e. Tianjin Union Medical Center, The First Affiliated Hospital of Nankai University, Tianjin, 300121, P. R. China

* Corresponding authors.

E-mail: wenfeidong@sibet.ac.cn (Wen-Fei Dong), zanmh@sibet.ac.cn (Minghui Zan)

† These authors contributed equally to this work.

**Experimental section**

**Chemicals and reagents**

N-[3-(Trimethoxysilyl) propyl] ethylenediamine (DAMO), 3-Aminopropyltriethoxysilane (APTES), 3-Aminopropyltrimethoxysilane (APTMS), 3-(2-Aminoethylamino)propyl-dimethoxymethylsilane (AEAPS), 9,10-diaminophenanthrene (910DAPT), fluorescein (Fluc), sulforhodamine 101 (SR101), Nile blue (NiB), paraformaldehyde, urea, and urea-formaldehyde resin were obtained from Titan Scientific (Shanghai, China).

**Characterization methods**

The surface observation of the materials was reported by JEOL- 2100F transmission electron microscope (TEM) with 200 kV acceleration voltage. Thermogravimetric Analysis and Differential Scanning Calorimetry (TG-DSC) were obtained by using STA 409PC (Netzsch, Germany). Raman spectra were obtained by using LabRAM HR Evolution. ^1^H nuclear magnetic resonance (NMR) spectra were obtained by using Varian 400 spectrometers. The X-ray photoelectron spectrometer (ESCALAB 250Xi spectrometer, Thermo Fisher) was used for collecting the X-ray photoelectron spectroscopy (XPS) spectra. X-ray diffraction (XRD) patterns were obtained on an X’Pert Pro diffractometer. The FT-IR spectrometer (Agilent Cary 660, United States) was selected to record the Fourier transform infrared (FT-IR) results. Phosphorescence spectra, phosphorescence lifetime, fluorescence lifetime, and fluorescent anisotropy were measured by a photoluminescence Spectrometer (FLS 1000, United Kingdom). Fluorescence excitation and emission spectra were acquired by the F97Pro FL spectrophotometer (Lengguang Technology, China). A U-3010 spectrophotometer (Hitachi, Japan) was used to analyze the UV absorption spectra of this experiment. Transient absorption spectroscopy was performed using a commercial femtosecond pump-probe system (Transient Absorption Spectrometer, Newport Corporation). Photographs of afterglow were taken using a FUJI Film (X - S10) under excitation by a handheld 365 nm UV lamp.

**Preparation of SiNDs**

2.0 mL DAMO and 15.0 mg of 910DAPT were dissolved in 20.0 mL of ultrapure water and sonicated for 20 minutes until completely dissolved. The mixed solution was then transferred to a hydrothermal reactor, and the reactor was placed in a desiccator and heated at 150°C for 5 hours. After the reaction was finished and cooled to room temperature, the crude product was loaded into a dialysis bag (MWCO: 1000), and the crude product was dialyzed and purified with ultrapure water as dialysate for 8 hours to remove impurities. Finally, the purified solution was freeze-dried, and the solid powder was collected.

**Preparation of SiNDs@U+F**

0.1 g of SiNDs was dissolved in 20 mL of ultrapure water, then 1.0 g of urea and 0.5 g of paraformaldehyde were added to the solution. After ultrasonication for 20 minutes, the mixed solution was transferred to a beaker, which was placed in a desiccator and heated at 150°C for 5 hours. After the reaction was finished and cooled to room temperature, the resulting solid was completely ground into powder. Finally, the product was washed three times with ultrapure water and anhydrous ethanol, respectively, and dried in an oven at 60°C for 1 h to obtain the final product.

**Preparation of Fluc/SiNDs@U+F with different weight concentrations**

(0.5/1.5/5.0 mg) of Fluc and 0.1 g of SiNDs were dissolved in 20 mL of ultrapure water, respectively, and 1.0 g of urea and 0.5 g of paraformaldehyde were added to the solution. After sonication for 20 minutes, the mixed solution was transferred to a beaker, which was heated in a desiccator at 150°C for 5 hours. After the reaction was completed and cooled to room temperature, the resulting solid was purified by the same purification method as for SiNDs@U+F to obtain the final product.

**Preparation of SR101/SiNDs@U+F with different weight concentrations**

(1.0/3.0/10.0 mg) of SR101 and 0.1 g of SiNDs were dissolved in 20 mL of ultrapure water, respectively, and 1.0 g of urea and 0.5 g of paraformaldehyde were added to the solution. After sonication for 20 minutes, the mixed solution was transferred to a beaker, which was heated in a desiccator at 150°C for 5 hours. After the reaction was completed and cooled to room temperature, the resulting solid was purified by the same purification method as for SiNDs@U+F to obtain the final product.

**Preparation of NiB/SiNDs@U+F with different weight concentrations**

(0.5/1.5/5.0 mg) of NiB, 0.1 g of SiNDs, and 10.0 mg SR101 were dissolved in 20 mL of ultrapure water, respectively, and 1.0 g of urea and 0.5 g of paraformaldehyde were added to the solution. After sonication for 20 minutes, the mixed solution was transferred to a beaker, which was heated in a desiccator at 150°C for 5 hours. After the reaction was completed and cooled to room temperature, the resulting solid was purified by the same purification method as for SiNDs@U+F to obtain the final product.

**Preparation of SiNDs@UF**

0.1 g of SiNDs was dissolved in 20 mL of ultrapure water, and 1.0 g of urea-formaldehyde resin was added to the solution. After ultrasonication for 20 minutes, the mixed solution was transferred to a beaker, which was placed in a desiccator and heated at 150°C for 5 hours. After the reaction was completed and cooled to room temperature, the resulting solid was purified by the same purification method as for SiNDs@U+F to obtain the final product.

**Preparation of SiNDs@U**

0.1 g of SiNDs was dissolved in 20 mL of ultrapure water, and 1.0 g of urea was added to the solution. After ultrasonication for 20 minutes, the mixed solution was transferred to a beaker, which was placed in a desiccator and heated at 150°C for 5 hours. After the reaction was completed and cooled to room temperature, the resulting solid was purified by the same purification method as for SiNDs@U+F to obtain the final product.

**Preparation of CDs**

15.0 mg of 9,10-diaminophenanthrene was dissolved in 20mL of ultrapure water and sonicated for 20 minutes until completely dissolved. After the same hydrothermal treatment and purification as for SiNDs, it was freeze-dried and the solid powder was collected.

**Preparation of CDs@U+F**

0.1 g of CDs was dissolved in 20 mL of ultrapure water, then 1.0 g of urea and 0.5 g of paraformaldehyde were added to the solution. After ultrasonication for 20 minutes, the mixed solution was transferred to a beaker, which was placed in a desiccator and heated at 150°C for 5 hours. After the reaction was completed and cooled to room temperature, the resulting solid was purified by the same purification method as for SiNDs@U+F to obtain the final product.

**Preparation of e@U**

1.0 g of urea was dispersed in 20 mL of ultrapure water and ultrasonicated for 20 minutes, then the mixed solution was transferred to a beaker, which was placed in a desiccator and heated at 150°C for 5 hours. After the reaction was completed and cooled to room temperature, the resulting solid was purified by the same purification method as for SiNDs@U+F to obtain the final product.

**Preparation of e@U+F**

1.0 g of urea and 0.5 g of paraformaldehyde were dispersed in 20 mL of ultrapure water and ultrasonicated for 20 minutes, then the mixed solution was transferred to a beaker, which was placed in a desiccator and heated at 150°C for 5 hours. After the reaction was completed and cooled to room temperature, the resulting solid was purified by the same purification method as for SiNDs@U+F to obtain the final product.

**Preparation of eUF**

1.0 g of urea-formaldehyde resin was dispersed in 20 mL of ultrapure water and ultrasonicated for 20 minutes, then the mixed solution was transferred to a beaker, which was placed in a desiccator and heated at 150°C for 5 hours. After the reaction was completed and cooled to room temperature, the resulting solid was purified by the same purification method as for SiNDs@U+F to obtain the final product.

**
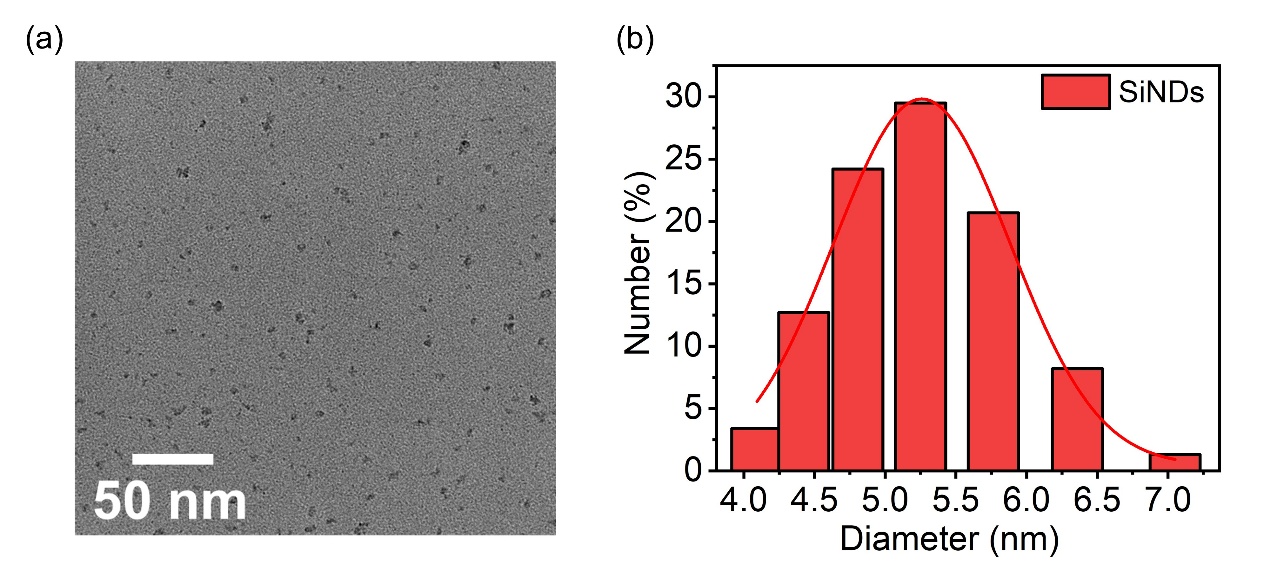
Figure S1**. TEM image (a) and size distribution (b) of SiNDs.

**
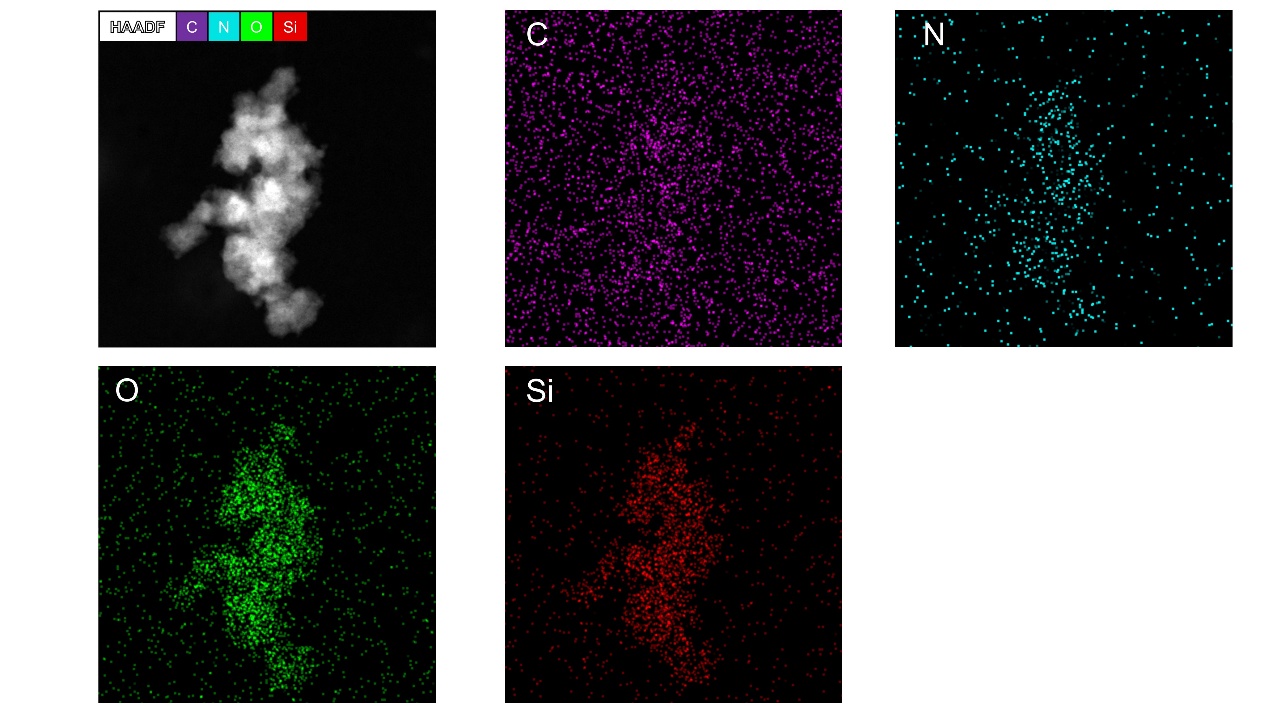
Figure S2**. Transmission electron microscopy energy-dispersive spectroscopy (TEM-EDS) images of SiNDs@U+F.

**
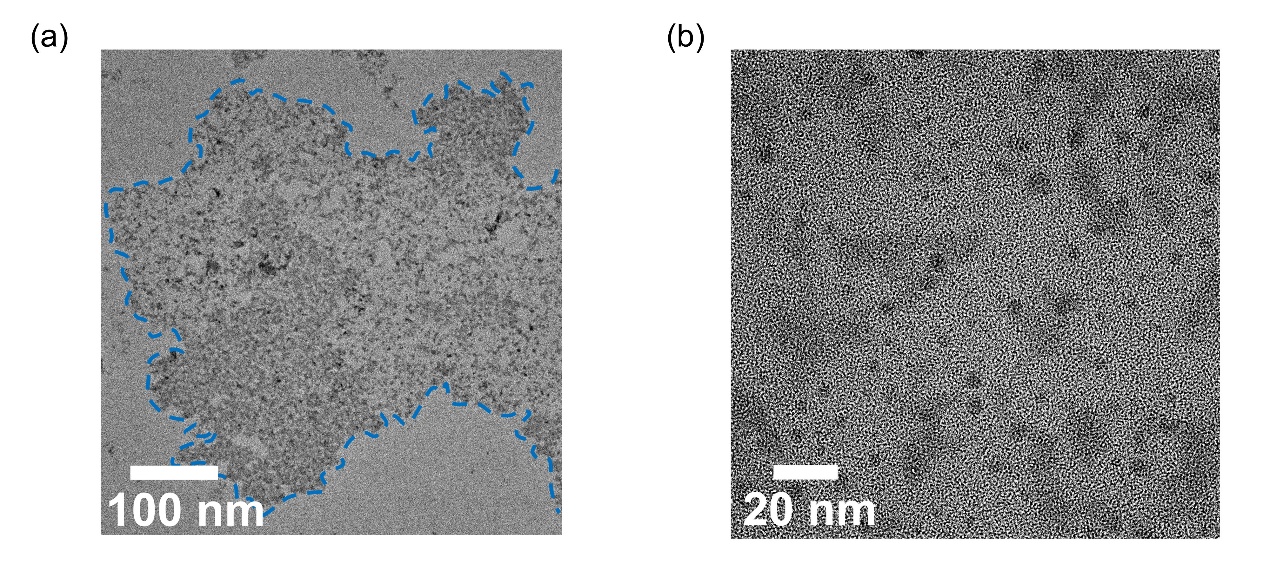
Figure S3**. TEM images of SiNDs@UF.

**
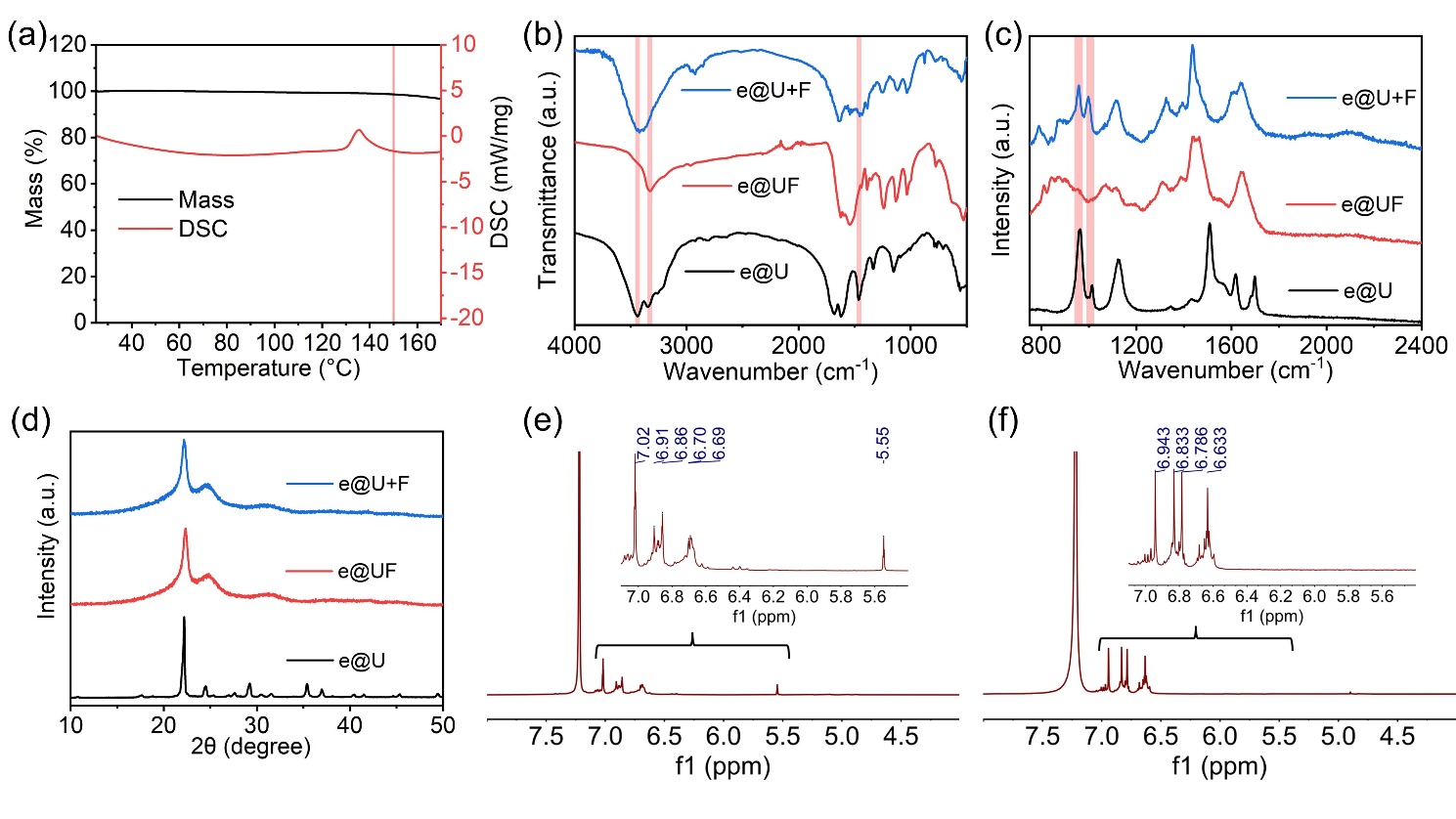
Figure S4**. (a) TGA and DSC curves for urea as a function of temperature under a N_2_ flow of 30 mL/min, at a heating rate of 5 °C/min. (b) FT-IR, (c) Raman, and (d) XRD spectra of e@U, e@UF, and e@U+F. The 1H NMR (151MHz, DCl) spectrum of (e) e@U+F and (f) e@UF.

**Figure S5**. Raman spectra of SiNDs@U and SiNDs@U+F.
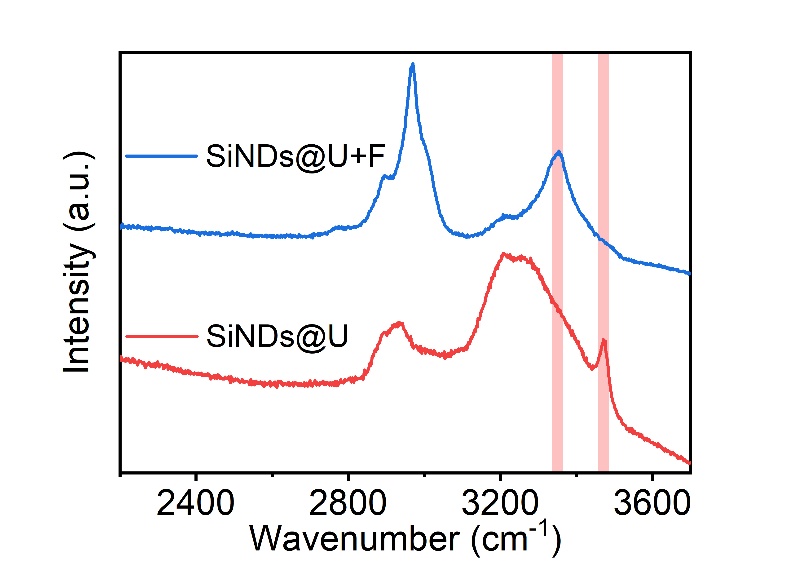


**
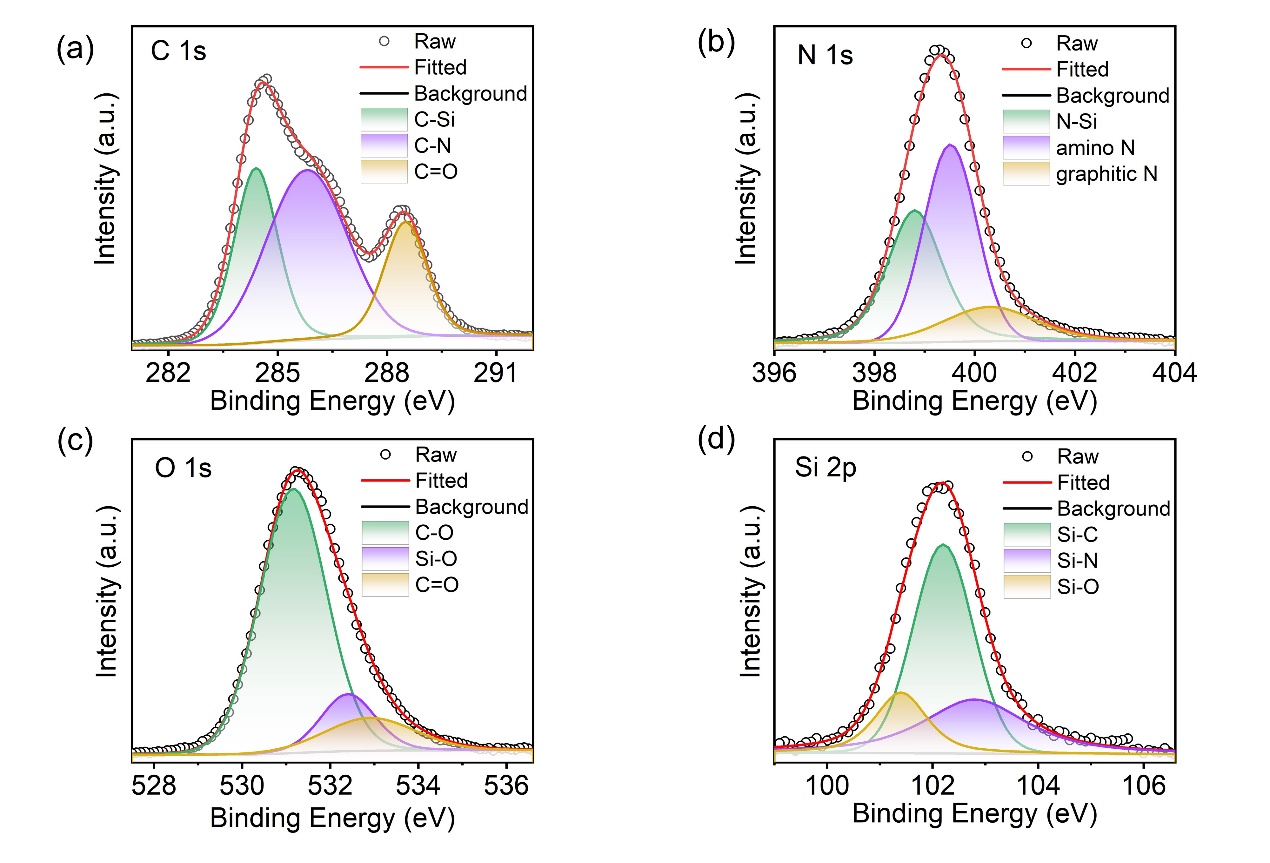
Figure S6**. High-resolution XPS spectra and the corresponding fitting curves of C 1s, N 1s, O 1s and Si 2p of SiNDs@U+F.

**Figure S7**. High-resolution XPS spectra and the corresponding fitting curves of C 1s, N 1s, O 1s and Si 2p of SiNDs.
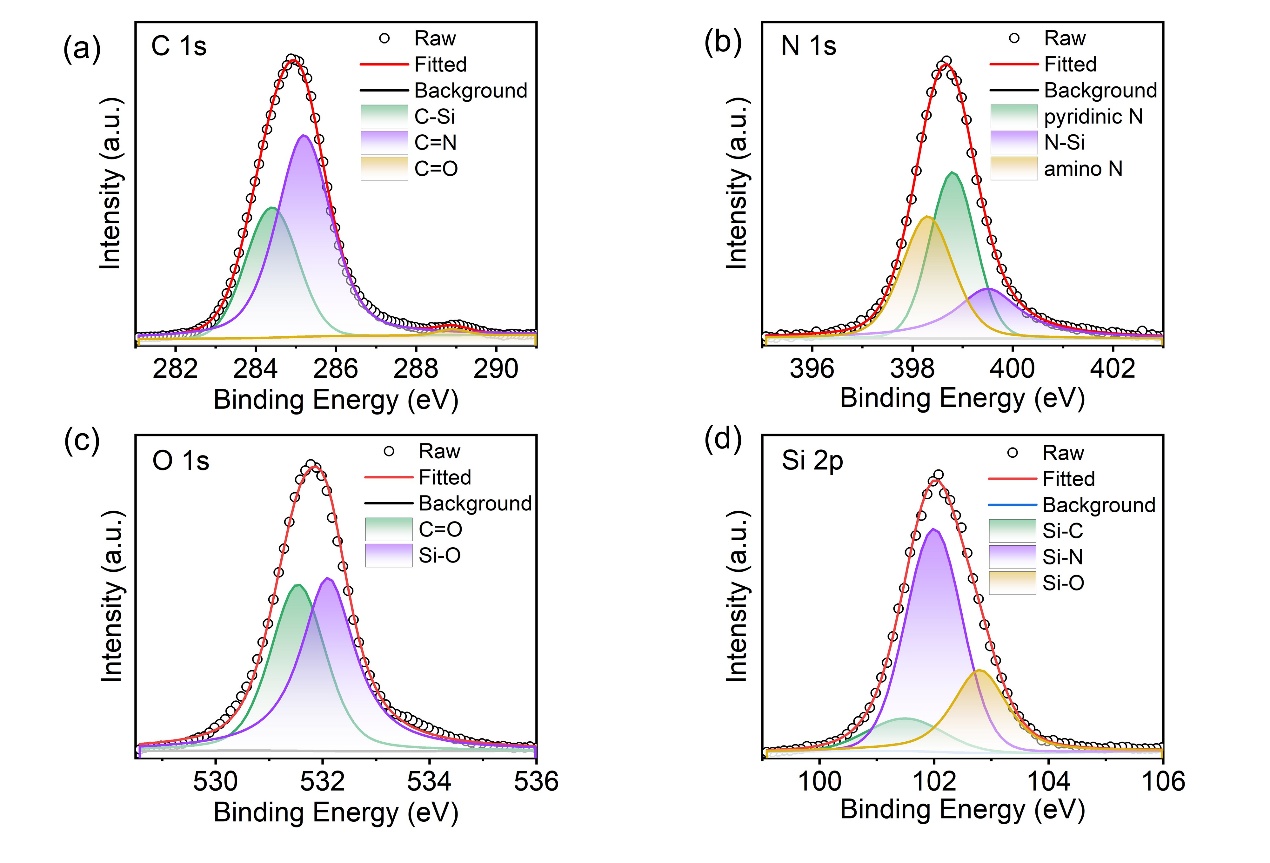


**Table S1.** High-resolution XPS fitting peaks of SiNDs.

| Elements | Content (%) | Figures | Bonds | Peaks (eV) |
| --- | --- | --- | --- | --- |
| C 1s | 56.01 | **Figure S7a** | C-Si | 284.4 |
|  |  |  | C-N | 285.8 |
|  |  |  | C=O | 288.5 |
| N 1s | 15.23 | **Figure S7b** | pyridinic N | 398.3 |
|  |  |  | N-Si | 398.8 |
|  |  |  | amino N | 399.5 |
| O 1s | 18.25 | **Figure S7c** | C=O | 531.5 |
|  |  |  | Si-O | 532.1 |
| Si 2p, Si 2s | 10.51 | **Figure S7d** | Si-C | 101.5 |
|  |  |  | Si-N | 102.0 |
|  |  |  | Si-O | 102.8 |

**Quantum yield measurements**

The absolute photoluminescence quantum yields (PLQY) of all solid-state compounds were measured using an Edinburgh FLS1000 spectrophotometer equipped with an integrating sphere under ambient conditions. The phosphorescence quantum yields (PhQY) of the materials were obtained from the following equation^[1]^:

*PhQY* = $\frac{B}{A+B}\times PLQY$

**
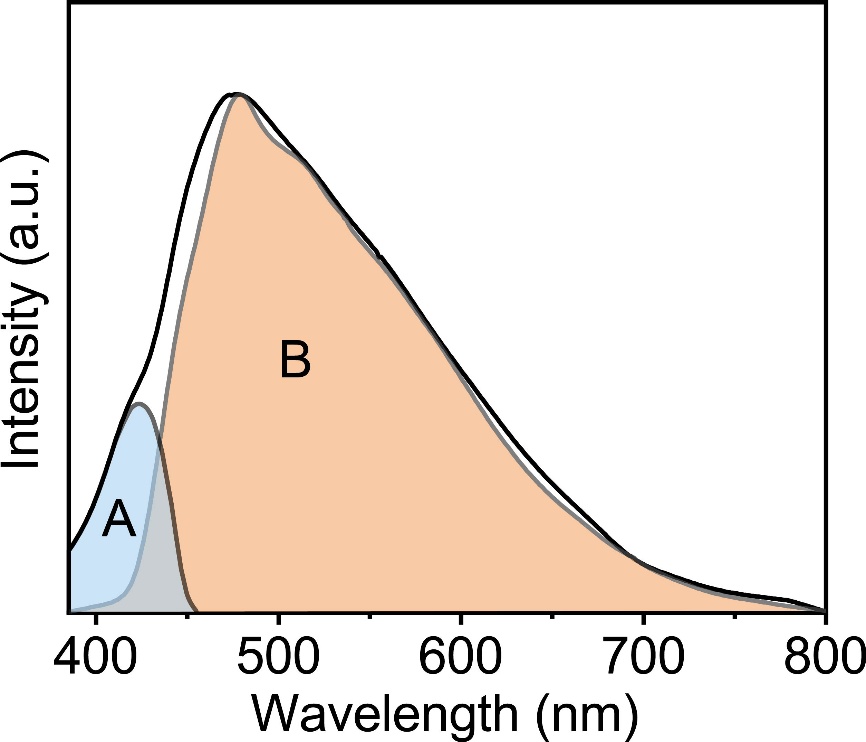
**Where A+B represents the total steady-state photoluminescence spectrum, A and B represent the integral areas of fluorescence and phosphorescence spectra, respectively. The phosphorescence was separated from PL in a time-gated scheme.

**Figure S8.** Schematic diagram of the integral area of photoluminescence and phosphorescence for quantum yield calculation.

**Table S2.** A comparison between the afterglow wavelength and phosphorescence lifetimes for the SiNDs@U+F composite with those afterglow materials.

| Afterglow  materials | Afterglow wavelength (nm) | Lifetimes  (s) | PhQY (%) | Reference |
| --- | --- | --- | --- | --- |
| TNP crystals | 454 | 0.18 | 96.50 | ^[1]^ |
| TSP crystals | 447 | 0.17 | 66.90 |  |
| DPP-BOH-PVA | 475 | 2.43 | 7.51 | ^[2]^ |
| p-Bp-BOH-PVA | 485 | 2.34 | 12.33 | ^[3]^ |
| BCZ-PVP | 486 | 4.18 | 22.40 | ^[4]^ |
| PAMCz | 414 | 4.20 | 20.70 | ^[5]^ |
| TP@PVA | 470 | 3.29 | 33.10 | ^[6]^ |
| Co-tCE | 560 | 2.03 | 36.50 | ^[7]^ |
| CZBA-PVA | 441 | 1.79 | 17.80 | ^[8]^ |
| PHA-PVA | 466 | 2.22 | 25.30 | ^[9]^ |
| 9HMF@PVA | 465 | 2.87 | 11.30 | ^[10]^ |
| PAC4 | 416 | 1.87 | 15.87 | ^[11]^ |
| PPU-0.35 | 447/478 | 1.89 | 18.50 | ^[12]^ |
| oP-CDs@B_2_O_3_ | 500 | 3.62 | 10.20 | ^[13]^ |
| TPZ-1/BPO | 461 | 0.29 | 75.28 | ^[14]^ |
| PXT/TX | 457 | 0.05 | 98.90 | ^[15]^ |
| N-CDs-Ⅱ | 512 | 0.88 | 81.13 | ^[16]^ |
| pP2Am@Mg(H_2_PO_4_)_2_ | 424 | 1.26 | 75.87 | ^[17]^ |
| pPCAAm@Mg(H_2_PO_4_)_2_ | 425 | 0.96 | 56.58 |  |
| pP2CA@Mg(H_2_PO_4_)_2_ | 427 | 0.84 | 45.33 |  |
| 1/CB[8] in filter paper | 620 | 0.11 | 99.38 | ^[18]^ |
| SiNDs@U+F | 476 | 3.44 | 81.04 | This work |

**
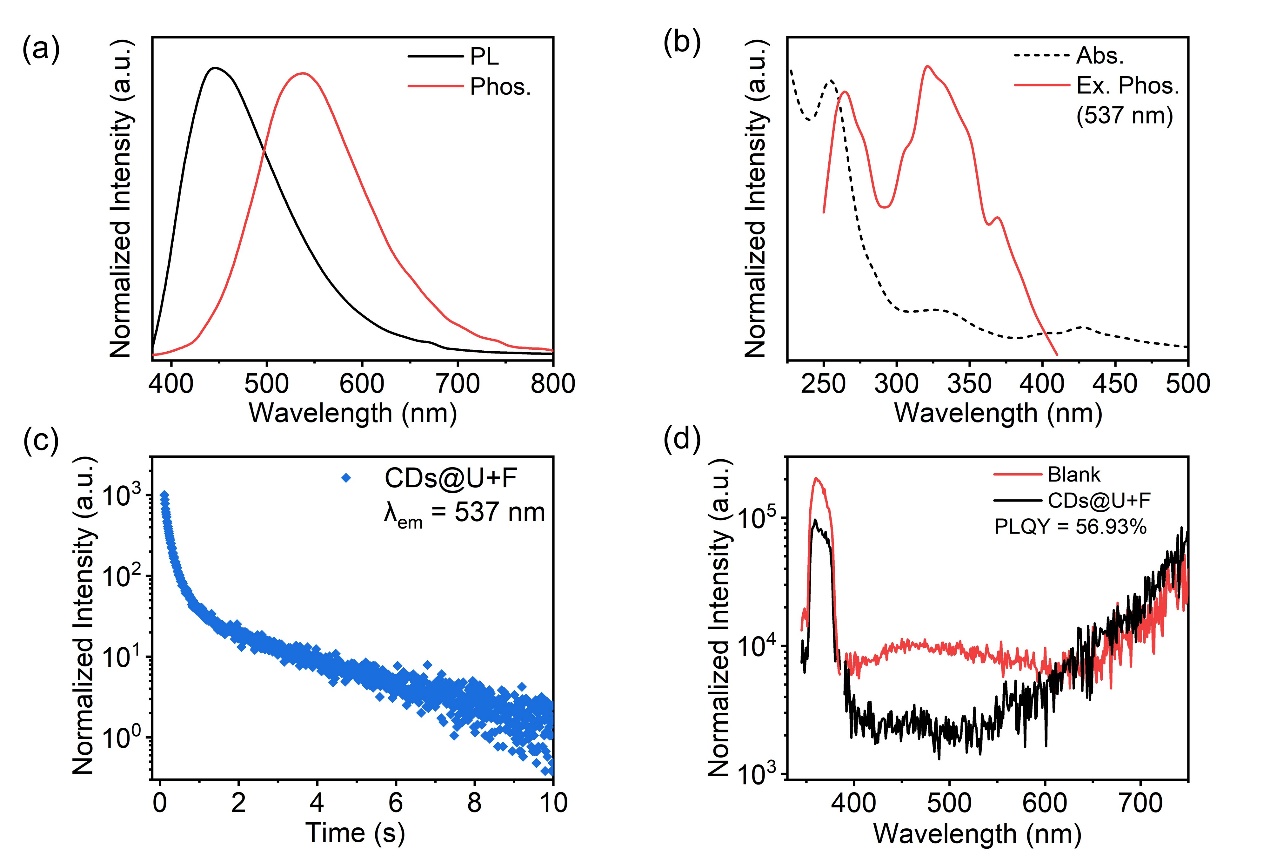
Figure S9**. (a) Steady-state photoluminescence and phosphorescence spectra of CDs@U+F under 365 nm excitation. (b) UV absorption spectrum of CDs@U+F, and excitation spectrum when phosphorescence emission is 510 nm. (c) Time-resolved afterglow decay curve and (d) PLQY of CDs@U+F under 365 nm excitation.

**Table S3.** Dynamic photophysical parameters of SiNDs@U+F

| τ_f_/ns | τ_p_/s | *Φ*_f_/% | *Φ*_p_/% | *Φ*_isc_/% | *k*_f,r_/s^-1^ | *k*_isc_/s^-1^ | *k*_p,r_/s^-1^ | *k*_p,nr_/s ^-1^ | *ΔE*_ST_/eV |
| --- | --- | --- | --- | --- | --- | --- | --- | --- | --- |
| 12.21 | 3.44 | 10.95 | 81.04 | 89.05 | 0.90🞨10^7^ | 7.29🞨10^7^ | 0.26 | 0.03 | 0.35 |

The dynamic photophysical parameters were calculated based on following equations^[2]^:

*k*_f,r_ = *Φ*_f_ /τ_f_

*Φ*_isc_ = 1-*Φ*_f_ -*Φ*_ic_ ≈ 1-*Φ*_f_

τ_f_ = 1/( *k*_f,r_ + *k*_f,nr_ + *k*_isc_)

*Φ*_isc_ = *k*_isc_ /( *k*_f,r_ + *k*_f,nr_ + *k*_isc_) = *k*_isc_ 🞨τ_f_

*k*_isc_ = *Φ*_isc_ /τ_f_

τ_p_ = 1/( *k*_p,r_ + *k*_p,nr_);

*Φ*_p_ = (*Φ*_isc_ 🞨 *k*_p,r_)/( *k*_p,r_ + *k*_p,nr_) = *Φ*_isc_ 🞨 *k*_p,r_ 🞨τ_p_

*k*_p,r_ = *Φ*_p_ /(*Φ*_isc_ 🞨τ_p_)

*k*_p,nr_ = 1/τ_p_ - *k*_p,r_

*E*_vert_ = 1240/λ

*ΔE*_ST_ = 1240/λ_FL_ - 1240/λ_Phos_

Where, *Φ, k*_f,r_ , *k*_isc_ , *k*_p,r_ , *k*_p,nr_, are the quantum yield, radiative rate constant of prompt fluorescence, rate constant of intersystem crossing (ISC), radiative rate constant of phosphorescence, nonradiative rate constant of phosphorescence.

**Table S4.** Photoluminescence properties of different SiNDs@U+F and CDs@U+F.

| Name | Precursors | Phos. peak (nm) | Lifetime (s) | PLQY (%) |
| --- | --- | --- | --- | --- |
| SiNDs@U+F | DAMO,910DAPT | 476 | 3.44 | 91.99 |
| CDs@U+F | 910DAPT | 537 | 1.68 | 56.93 |
| aSiNDs@U+F | APTES,910DAPT | 482 | 3.08 | 86.10 |
| bSiNDs@U+F | APTS,910DAPT | 495 | 3.22 | 84.58 |
| cSiNDs@U+F | AEEA,910DAPT | 507 | 3.16 | 83.64 |

**Figure S10.** (a) Phosphorescence spectra and (b) Time-resolved phosphorescence decay curves of aSiNDs@U+F, bSiNDs@U+F and cSiNDs@U+F under 365 nm excitation. PLQY of (c) aSiNDs @U+F, (d) bSiNDs @U+F, and (e) cSiNDs @U+F under 365 nm excitation.**
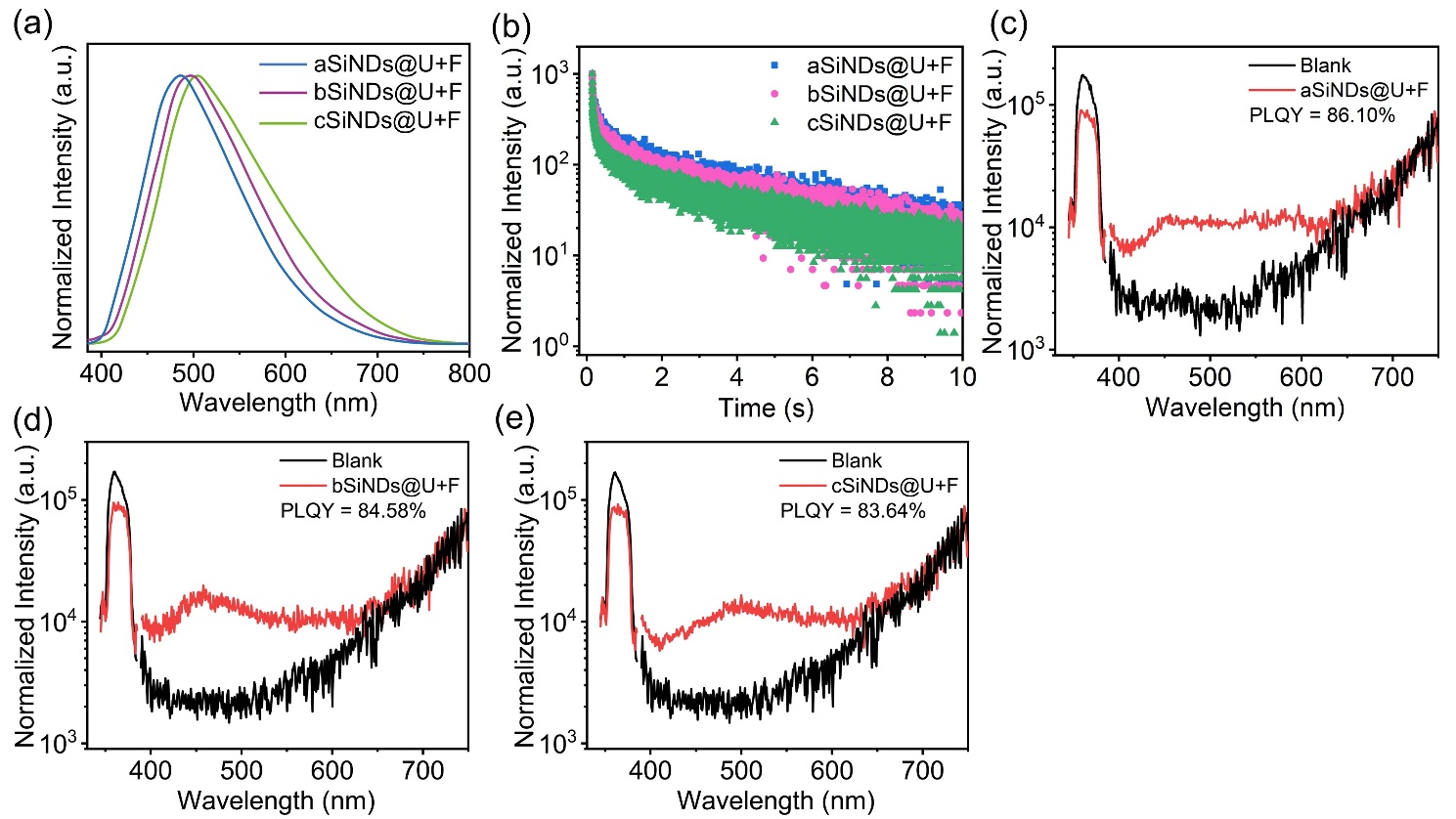
**


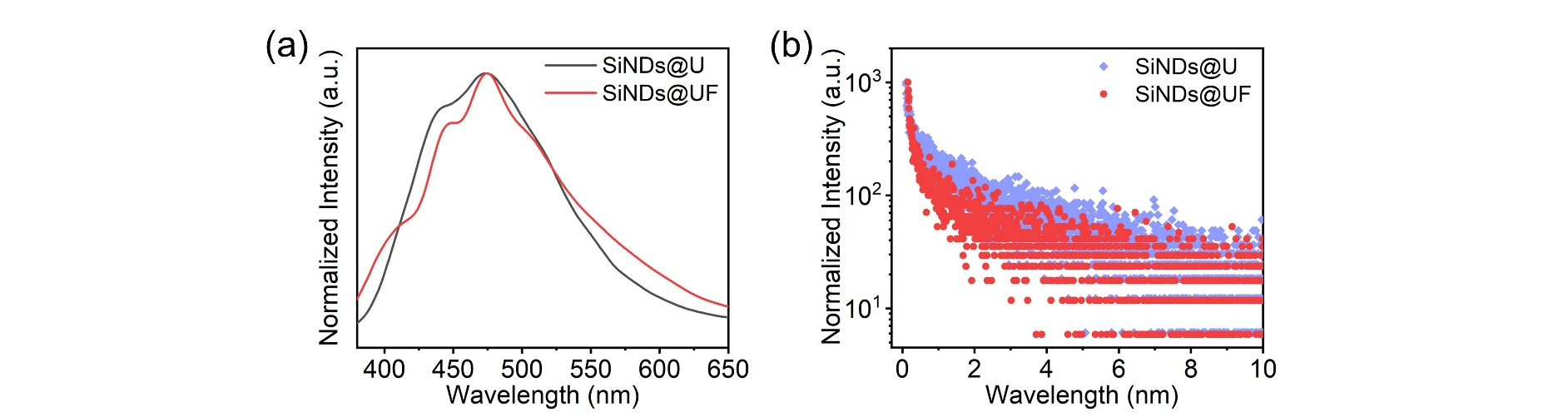
**Figure S11**. (a) Phosphorescence spectra and (b) Time-resolved afterglow decay curve of SiNDs@UF and SiNDs@U under 365 nm excitation.

**Figure S12.** The phosphorescence stability of SiNDs@U+F, SiNDs@UF, and SiNDs@U under (a) nitrogen, (b) ambient air, (c) aqueous environments, (d) oxidizing agents and organic solvents.
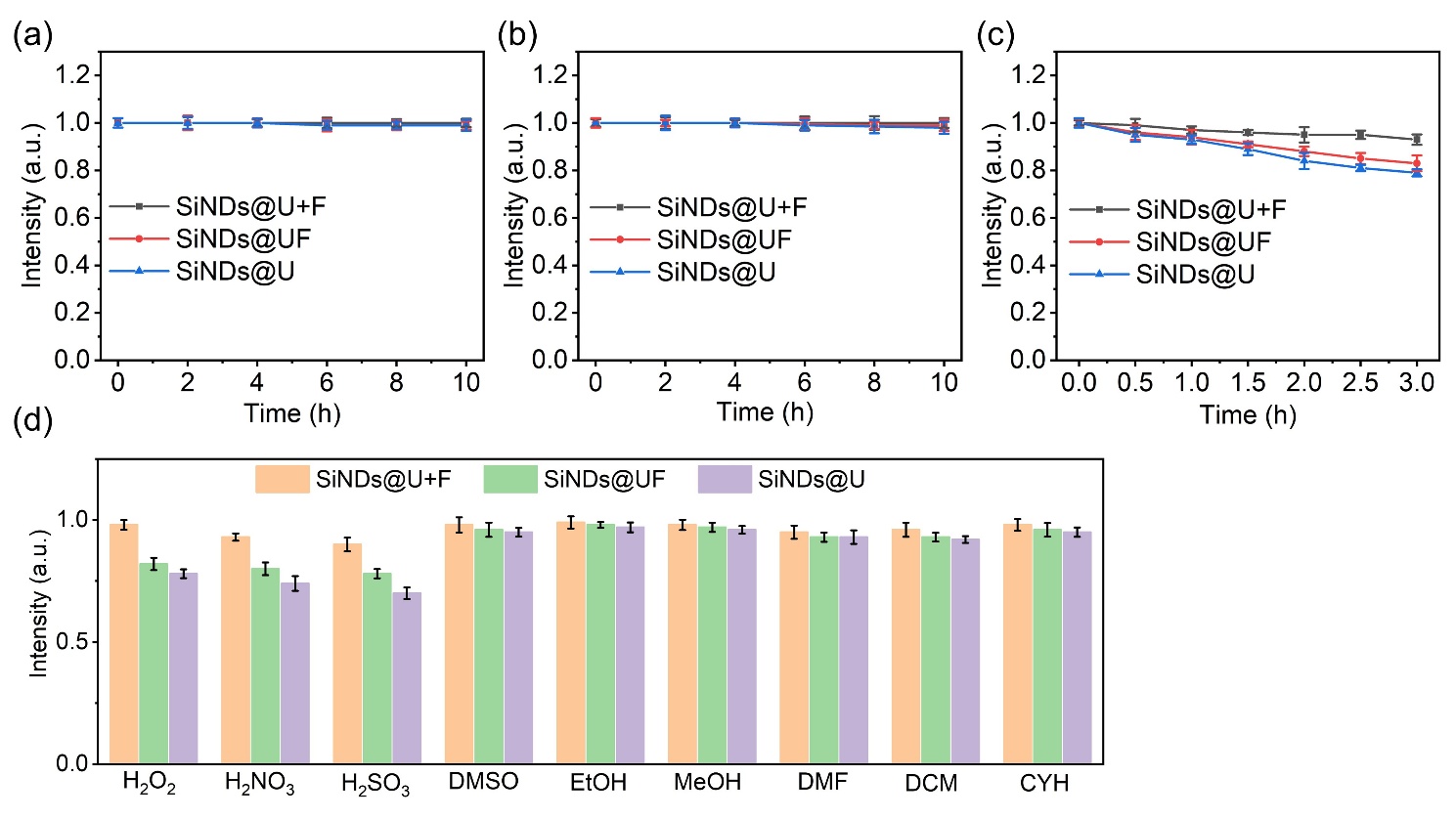
 (n = 3).

**
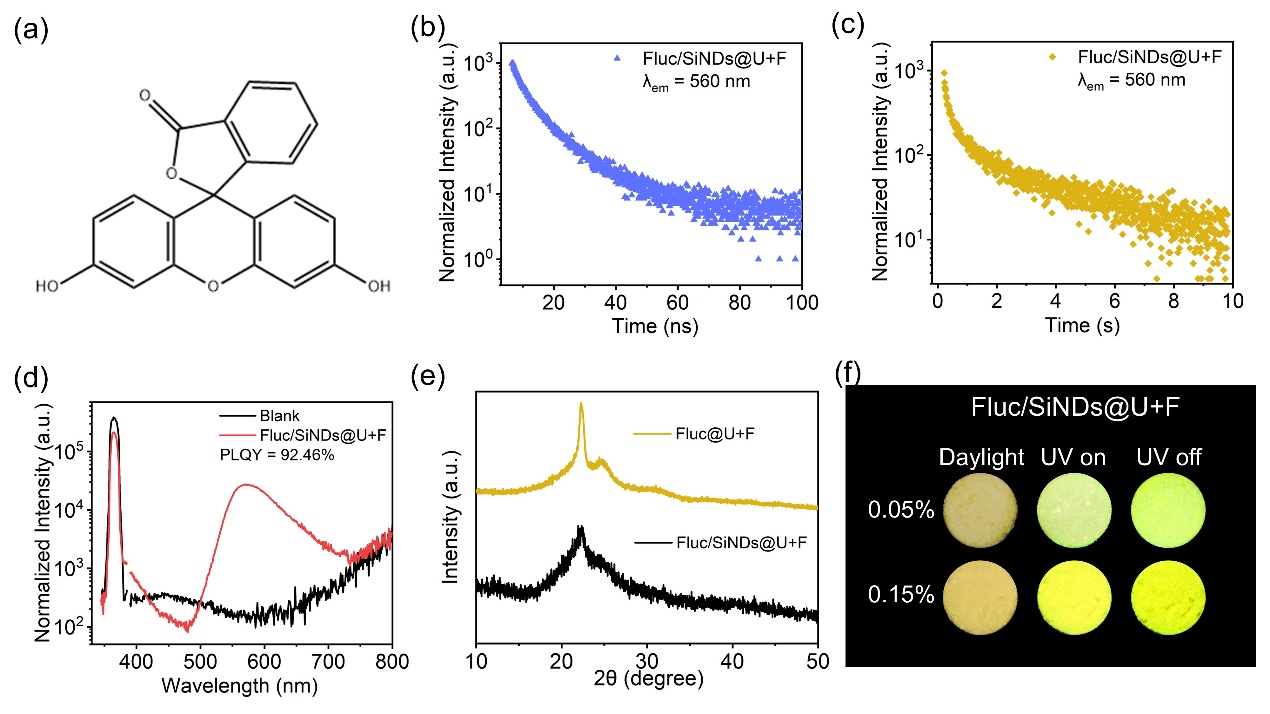
Figure S13**. (a) The structural formula of fluorescein. (b) Time-resolved fluorescence and (c) afterglow decay curves of Fluc/SiNDs@U+F with 365 nm excitation. (d) PLQY of Fluc/SiNDs@U+F with 365 nm excitation. (e) XRD image of Fluc/SiNDs@U+F and Fluc@U+F. (f) Images of Fluc/SiNDs@U+F with different acceptor doping weight concentration under daylight, 365 nm UV lamp on and off.

**
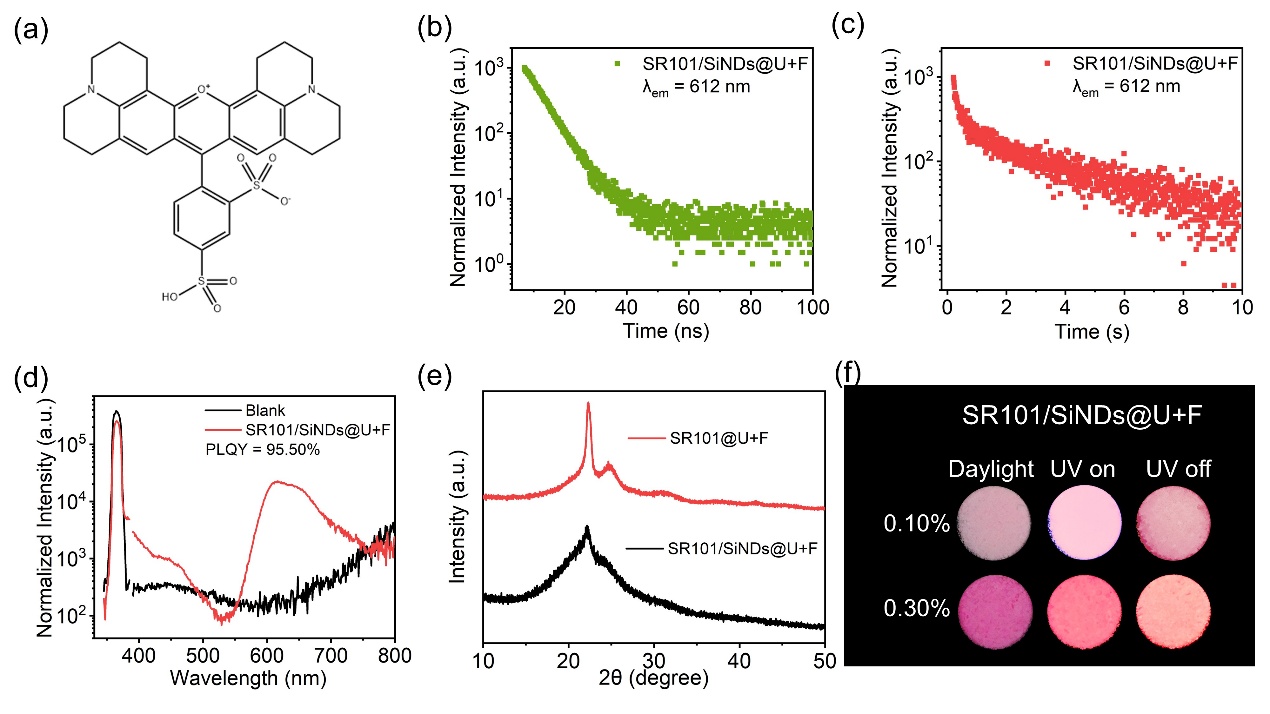
Figure S14**. (a) The structural formula of sulforhodamine 101. (b) Time-resolved fluorescence and (c) afterglow decay curves of SR101/SiNDs@U+F with 365 nm excitation. (d) PLQY of SR101/SiNDs@U+F with 365 nm excitation. (e) XRD image of SR101/SiNDs@U+F and SR101@U+F. (f) Images of SR101/SiNDs@U+F with different acceptor doping weight concentration under daylight, 365 nm UV lamp on and off.

The anisotropy (*r*) was calculated using the following equation^[19]^:

*r* = $\frac{I_{VV} - GI_{VH}}{I_{VV} + 2GI_{VH}}$


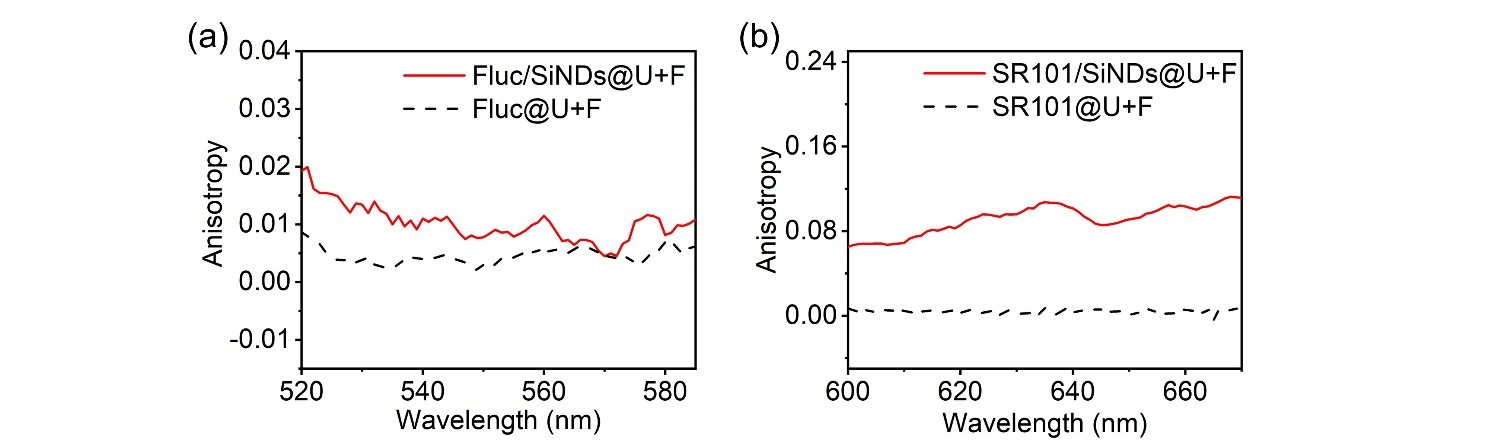
Where *I*_VH_ is fluorescent intensity with a vertical excitation polarizer and a horizontal emission polarizer, *I*_VV_ is fluorescent intensity with vertical excitation and emission polarizers, and *G* is the correction factor of the instrument (*G* = *I*_VH_/*I*_VV_).

**Figure S15.** (a) Fluorescent anisotropy (*r*) values of Fluc/SiNDs@U+F and Fluc@U+F. (b) Fluorescent anisotropy (*r*) values of SR101/SiNDs@U+F and SR101@U+F.

**
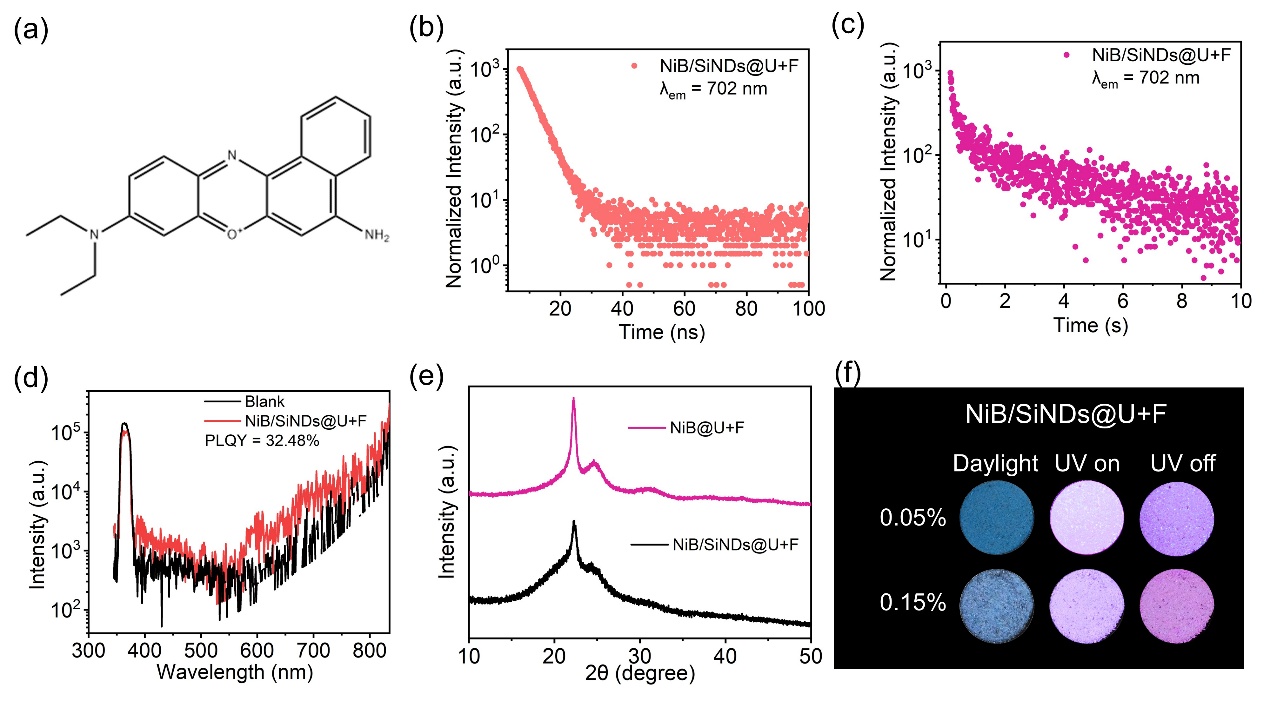
Figure S16**. (a) The structural formula of Nile blue. (b) Time-resolved fluorescence and (c) afterglow decay curves of NiB/SiNDs@U+F with 365 nm excitation. (d) PLQY of NiB/SiNDs@U+F with 365 nm excitation. (e) XRD image of NiB/SiNDs@U+F and NiB@U+F. (f) Images of NiB/SiNDs@U+F with different acceptor doping weight concentration under daylight, 365 nm UV lamp on and off.

**Table S5.** The resonance energy transfer efficiency of three systems.

| Name | τ_D0_ (s) | τ_D_ (s) | Φ_ET_ (%) |
| --- | --- | --- | --- |
| Fluc/SiNDs@U+F | 3.44 | 0.55 | 84.01 |
| SR101/SiNDs@U+F | 3.44 | 0.35 | 89.88 |
| NiB/SiNDs@U+F | 2.51 | 0.19 | 92.43 |

The resonance energy transfer efficiency (Φ_ET_) between the energy donor and acceptor calculated from the following equation^[20]^:

Φ_ET_ = 1 - $\frac{\tau_{D}}{\tau_{D0}}$

Where τ_D_ is the lifetime of the donor in the presence of an acceptor, and τ_D0_ is the lifetime of the donor in the absence of an acceptor.

**Figure S17**. A flower anti-counterfeiting image composed of four materials. (b) Time-resolved fluorescence and (c)
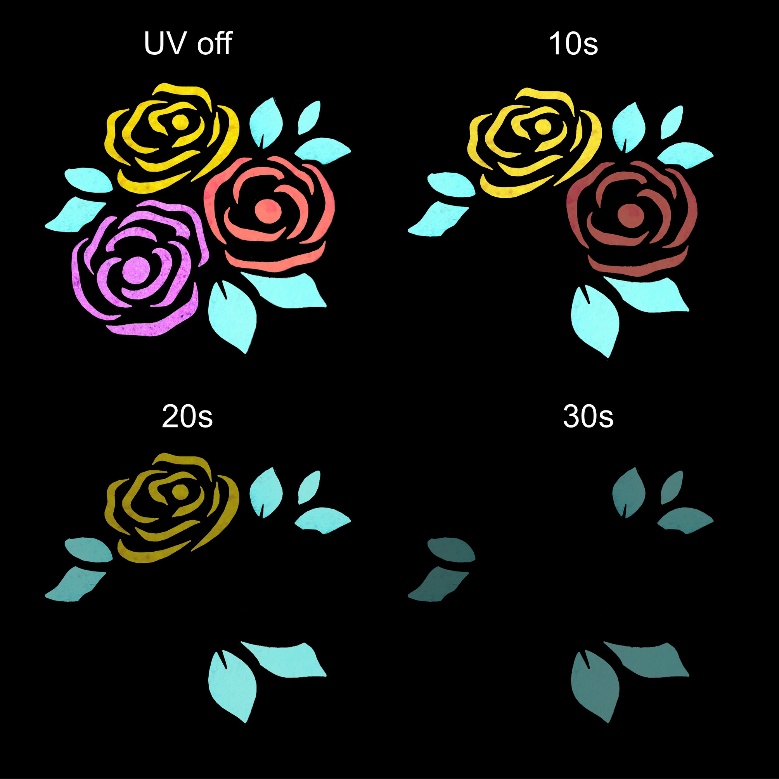


**
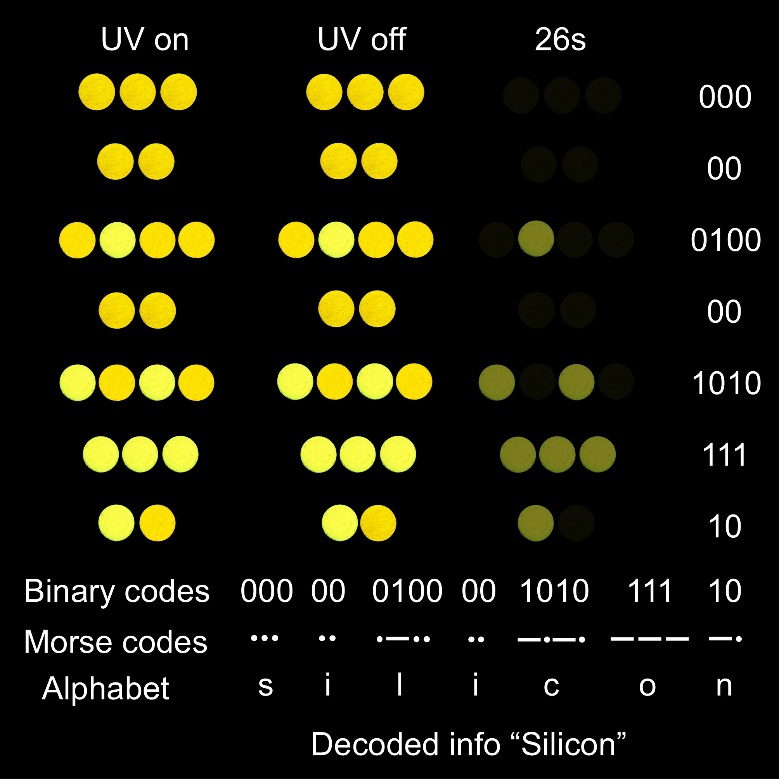
Figure S18**. Application demonstration of information decoding after turning off UV lamp excitation.

**Figure S19**. After different decay times, the afterglow intensity and corresponding letter symbols
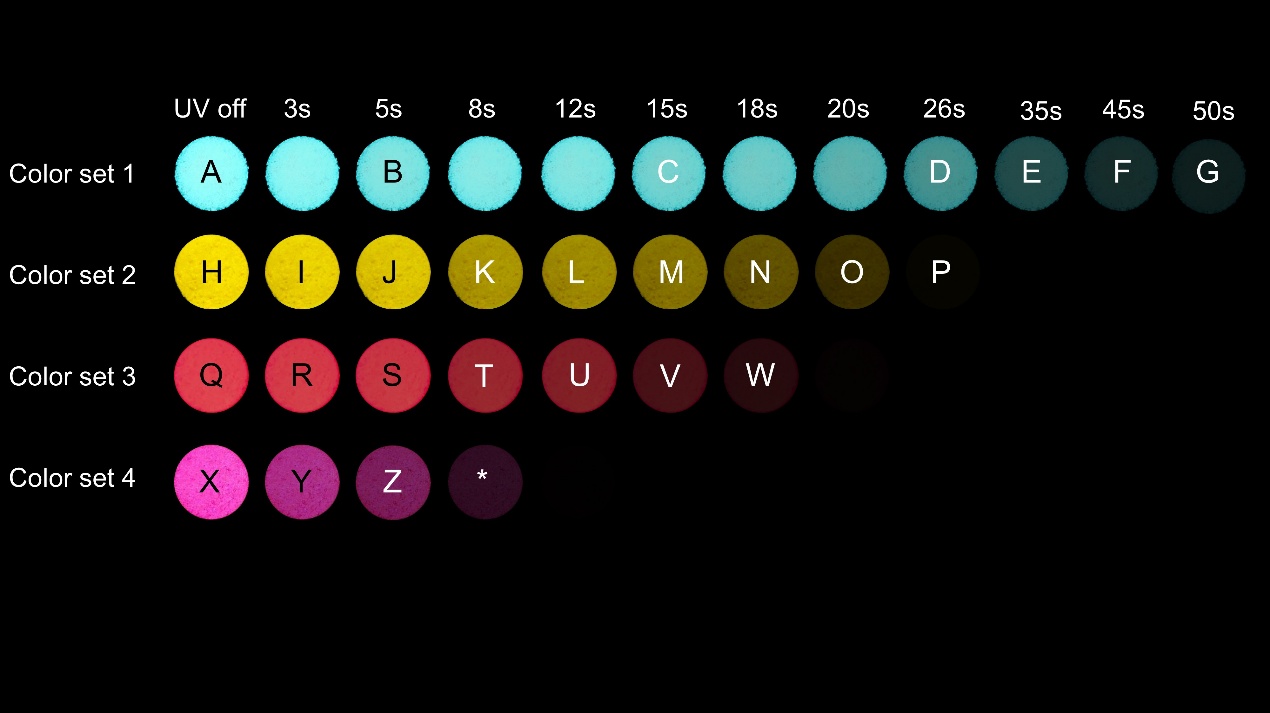


**References**

[1] W. Ye, H. Ma, H. Shi, H. Wang, A. Lv, L. Bian, M. Zhang, C. Ma, K. Ling, M. Gu, Y. Mao, X. Yao, C. Gao, K. Shen, W. Jia, J. Zhi, S. Cai, Z. Song, J. Li, Y. Zhang, S. Lu, K. Liu, C. Dong, Q. Wang, Y. Zhou, W. Yao, Y. Zhang, H. Zhang, Z. Zhang, X. Hang, Z. An, X. Liu, W. Huang, *Nat. Mater.* **2021**, *20*, 1539-1544.

[2] D. Li, Y. Yang, J. Yang, M. Fang, B. Z. Tang, Z. Li, *Nat. Commun.* **2022**, *13*, 347.

[3] D. Li, J. Yang, M. M. Fang, B. Z. Tang, Z. Li, *Sci. Adv.* **2022**, *8*, eabl8392

[4] K. Chen, Y. Zhang, Y. Lei, W. Dai, M. Liu, Z. Cai, H. Wu, X. Huang, X. Ma, *Nat. Commun.* **2024**, *15*, 1269.

[5] H. Peng, G. Z. Xie, Y. Cao, L. Y. Zhang, X. Yan, X. Zhang, S. H. Miao, Y. Tao, H. H. Li, C. Zheng, W. Huang, R. F. Chen, *Sci. Adv.* **2022**, *8*, eabk2925.

[6] F. Lin, H. Wang, Y. Cao, R. Yu, G. Liang, H. Huang, Y. Mu, Z. Yang, Z. Chi, *Adv. Mater.* **2022**, *34*, 2108333.

[7] J. Yu, Z. Sun, H. Ma, C. Wang, W. Huang, Z. He, W. Wu, H. Hu, W. Zhao, W. H. Zhu, *Angew. Chem. Int. Ed.* **2023**, *62*, e202316647.

[8] Y. Zhang, J. Chen, Q. Sun, H. Zhang, S. Xue, W. Yang, *Chem. Eng. J.* **2023**, *452*, 139385.

[9] H. Yang, Y. Wang, X. Yao, H. Ma, J. Yu, X. Li, X. Wang, X. Liang, Q. Peng, S. Cai, Z. An, W. Huang, *J. Am. Chem. Soc.* **2024**, *147*, 1474-1481.

[10] H. Hou, H. Wang, M. He, Q. Li, X. Wang, F. Guo, Q. Chen, L. Qu, C. Yang, *Angew. Chem. Int. Ed.* **2024**, *63*, e202411323.

[11] Y. Ding, C. Yang, F. Gan, G. Zhang, C. Shen, H. Qiu, *J. Am. Chem. Soc.* **2024**, *146*, 25211-25220.

[12] M. Chen, B. Liu, J. Ren, C. Zhang, Z. Ren, Z. H. Guan, *Adv. Mater.* **2025**, *37*, 2504825.

[13] Q. Zhang, S. Xu, L. Zhang, L. Yang, C. Jiang, *Adv. Sci.* **2024**, *11*, 2400781.

[14] L. Zhang, J. Li, Y. Zhang, W. Dai, Y. Zhang, X. Gao, M. Liu, H. Wu, X. Huang, Y. Lei, D. Ding, *Nat. Commun.* **2025**, *16*, 3970.

[15] H. Ma, L. Fu, X. Yao, X. Jiang, K. Lv, Q. Ma, H. Shi, Z. An, W. Huang, *Nat. Commun.* **2024**, *15*, 3660.

[16] M. You, C. Li, Z. Zhang, Y. Zhang, W. Li, X. Zhang, J. Zhuang, C. Hu, H. Dong, Y. Liu, B. Lei, M. Zheng, *Chem. Eng. J.* **2025**, *505*, 159246.

[17] T.-Y. Zhou, Q. Zhao, H.-Y. Zhao, C. Li, F.-Y. Zeng, J. Zhou, J. Li, Y. Wang, G.-Y. Li, Y. Liang, Y. Song, S.-H. Li, *Chem. Eng. J.* **2025**, *511*, 161969.

[18] X. K. Ma, W. Zhang, Z. Liu, H. Zhang, B. Zhang, Y. Liu, *Adv. Mater.* **2021**, *33*, 2007476.

[19] L. Colvin, D. Al Husseini, D. Tu, D. Dunlap, T. Lalonde, M. Üçüncü, A. Megia-Fernandez, M. Bradley, W. Liu, M. A. Grunlan, G. L. Coté, *ACS Sensors* **2024**, *9*, 6218-6227.

[20] D. Wang, J. Gong, Y. Xiong, H. Wu, Z. Zhao, D. Wang, B. Z. Tang, *Adv. Funct. Mater.* **2022**, *33*, 2208895.
